# Highly Resolved Community Sewage Metagenomics Unveiling Landscape and Transmission Patterns of Antibiotic Resistome in Hong Kong Populations

**DOI:** 10.1002/advs.202508389

**Published:** 2026-02-27

**Authors:** Jiahui Ding, Mengying Wang, Xiaoqing Xu, Dou Wang, Xi Chen, Shuxian Li, Xiawan Zheng, You Che, Yu Deng, Tommy T. Y. Lam, Liguan Li, Tong Zhang

**Affiliations:** ^1^ Environmental Microbiome Engineering and Biotechnology Lab Center For Environmental Engineering Research Department of Civil Engineering The University of Hong Kong Hong Kong SAR China; ^2^ HKU‐Pasteur Research Pole The University of Hong Kong Hong Kong SAR China; ^3^ Faculty of Dentistry The University of Hong Kong Hong Kong SAR China; ^4^ State Key Laboratory of Emerging Infectious Diseases School of Public Health The University of Hong Kong Hong Kong SAR China; ^5^ Centre For Immunology & Infection Hong Kong SAR China; ^6^ The Hong Kong Jockey Club Global Health Institute Hong Kong SAR China; ^7^ Department of Science and Environmental Studies The Education University of Hong Kong Hong Kong SAR China; ^8^ School of Public Health The University of Hong Kong Hong Kong SAR China; ^9^ Department of Environmental Science and Engineering Macau University of Science and Technology Macao Hong Kong SAR China; ^10^ State Key Laboratory of Marine Environmental Health City University of Hong Kong Hong Kong SAR China; ^11^ Shenzhen Institute of Research and Innovation The University of Hong Kong Shenzhen China

**Keywords:** antimicrobial resistance, ARG mobility, genetic context, host tracking, nanopore sequencing, sewage surveillance

## Abstract

The increasing global burden of antimicrobial resistance (AMR) has been identified as a critical public health crisis, necessitating the development of robust, real‐time surveillance frameworks to evaluate AMR dynamics. Sewage surveillance is emerging as a promising tool that utilizes sewage fingerprinting to provide comprehensive and unbiased information on antibiotic resistance genes (ARGs) within human populations. Here, we conducted a large‐scale, year‐long field surveillance of resistome in the community sewage using both short‐ and long‐read metagenomic sequencing. We examined samples collected from 95 geographically distributed sites across Hong Kong, covering a population of 4.8 million residents, during summer and winter seasons. Our findings revealed distinct seasonal patterns through high‐resolution resistome profiling. We found that the resistome structures shifted from the community sewage collected at sewer manholes to the influent of wastewater treatment plants (WWTPs), driven by taxonomic variation. Notably, community sewage exhibited a significantly higher similarity to the resistome of human feces than WWTP influent, which provides insights for selecting suitable sampling sites for epidemiological ARG surveillance. The application of long‐read sequencing markedly enhanced our understanding of the phylogenetic diversity of ARG hosts and uncovered a broad spectrum of potentially mobile ARGs with varied genetic backgrounds. Furthermore, we observed multiple local ARG transmission patterns and subsequently evaluated their potential threats to public health based on the gene trees to inform future epidemiological control strategies. Overall, this work expands our current understanding of community sewage for population‐level AMR monitoring and establishes a baseline for advancing sewage surveillance efforts to better combat AMR.

## Graphical abstract

A large‐scale, high‐resolution antimicrobial resistance (AMR) surveillance in Hong Kong community sewage combining short‐ and long‐read sequencing. The resistome profile is comprehensively characterized, and this upstream sampling strategy demonstrated superiority in reflecting the human gut resistome. The genetic contexts of AMR genes and their local transmission patterns are further investigated, expanding our current understanding of community sewage for population‐level AMR monitoring.

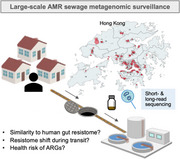

## 1 Introduction

Antimicrobial resistance (AMR) has become one of the major global public health concerns in the 21st century. The emergence of antibiotic resistance genes (ARGs) in bacteria via mutation [1] or horizontal gene transfer (HGT) [2], especially in clinical pathogens, contributes to rising rates of therapeutic failure in antimicrobial treatments for infectious diseases. It is estimated that there could be 1.91 million AMR‐attributable and 8.22 million AMR‐associated deaths annually by 2050 [3]. Apart from clinical settings, the widespread occurrence of ARGs in domestic and natural environments further exacerbates the threats posed by AMR [4]. The elevated magnitude of this challenge highlights the imperative to strengthen AMR mitigation strategies and safeguard public health, with surveillance serving as a cornerstone for elucidating transmission dynamics, assessing containment measures, and informing therapeutic choices [5]. The political declaration that has been approved in the United Nations General Assembly (UNGA) High‐Level Meeting on AMR of 2024 also encourages countries to report quality‐assured AMR surveillance data [6].

Current AMR surveillance largely relies on clinical surveillance systems that employ individual‐level sampling of prioritized human pathogens through phenotypic and genotypic analysis. However, this approach could introduce inherent selection biases, favoring populations with healthcare access, while neglecting the extensive reservoir of ARGs harbored by commensal bacterial communities. In contrast, sewage surveillance has gained prominence as a scalable, cost‐effective, and noninvasive strategy to obtain population‐level AMR data, as sewage samples are deemed aggregate collections of all residents in the specific sewershed catchment and are expected to provide an integrated view of AMR for both healthcare‐ and community‐associated populations [7]. Its utility is evidenced by successful applications in tracking pathogens, such as SARS‐CoV‐2 [8], influenza virus [9], mpox virus [10], and *Salmonella* [11], with strong correlations to clinical infection data. The COVID‐19 pandemic further accelerated methodological advancements and global infrastructure development for wastewater‐based epidemiology [12, 13, 14, 15], providing an impetus for scalable AMR surveillance at municipal, national, regional, and global levels.

Compared to conventional molecular‐based methods of AMR surveillance, such as quantitative polymerase chain reaction (qPCR) that focus on specific target genes, shotgun metagenomic sequencing shows superiority with the capacity to holistically characterize microbial communities and detect a wide spectrum of ARGs and pathogens present in samples without prior target selection [16]. Metagenomic sequencing has been successfully adopted to reveal significant findings regarding the resistance burden in urban populations, mainly based on short‐read sequencing [17, 18, 19, 20, 21, 22]. For example, it was reported that the sewage resistomes were stratified by geographic location [17, 18] and could reflect socioeconomic status better than the fecal resistome [19]. Additionally, core resistomes were detected in different population‐sourced sewage [20], and ARG abundance was found to correlate with antibiotic concentrations [21] and hospital antibiotic prescription levels [22]. However, most of these studies were conducted through the survey of untreated influent samples collected from inlets of municipal wastewater treatment plants (WWTPs), i.e., the end of the sewage collection system. Sampling at upstream sites instead of at the end of sewage conveyance is expected to reflect the resistance burden in the population in a better way and be more informative for pinpointing local hotspots and detecting the early emergence of ARGs for informed interventions. A demonstration of such high‐resolution surveillance is therefore needed. Moreover, the way in which the resistome, particularly human‐associated ARGs, shifts during sewage conveyance from residential buildings to WWTP in the sewage collection system remains unclear. Gaining insight into these dynamics would improve the interpretation of sewage surveillance data.

Furthermore, second‐generation short‐read sequencing faces challenges in the contextualization of ARGs regarding their host species and their co‐occurrence patterns with mobile genetic elements (MGEs). The genetic context is essential for assessing the health risk of ARGs [23]. For example, ARGs carried by pathogenic hosts may pose greater threats to human health, and those associated with MGEs may have higher transfer potential from commensal bacteria to human pathogens [24]. Although assembly could retrieve genes in proximity, it is always confounded by the presence of repetitive genomic regions [25]. The gaps above could be filled by third‐generation sequencing technologies, such as Oxford Nanopore sequencing [26] and PacBio sequencing [27], which are capable of generating reads spanning several to hundreds of kilobases. These platforms provide opportunities for direct investigation of the genetic context of ARGs, yet their applications in environmental surveillance remain limited.

In the present study, we systematically characterized the resistome of the community sewage collected from manholes at 95 sampling sites of the territory‐wide sewage surveillance system across Hong Kong using both short‐read (Illumina) and long‐read (Oxford Nanopore) metagenomic sequencing. The abundance and diversity of ARGs in communities of 4.8 million population, as well as their spatial and seasonal variance, were investigated. A significant resistome shift was observed along the transit of sewage, and the contribution of human feces to the community sewage resistome was found to be significantly higher than to the downstream influent resistome of WWTPs. Long‐read sequencing enabled high‐resolution characterization of ARG genomic contexts, uncovering novel ARG‐host associations and their co‐occurrence patterns with MGEs. Moreover, by analyzing polished long‐read assembled contigs, we inferred the local transmission status of clinically relevant ARGs from their phylogenetic diversity and their genetic context, and subsequently assessed their dissemination potential. We aimed to use this large‐scale AMR metagenomic surveillance with high spatial resolution (95 sites) and sequencing depth (2.2 Tb clean short‐read and 2.8 Tb clean long‐read data) to enhance our comprehension of ARG transmission and risk assessment and complement the monitoring efforts for better mitigation of AMR.

## 2 Results

### 2.1 Summary of Samples and Metagenomic Dataset

The 190 community sewage samples collected from 95 sites covering both the summer and winter seasons was subjected to metagenomic sequencing on both Illumina NovaSeq6000 and Oxford Nanopore PromethION platforms, generating 2.2 Tb of clean short‐read data (average sequencing depth: 11.8 Gb per sample) and 2.8 Tb of clean long‐read data (average sequencing depth: 14.5 Gb per sample) (Table S1). Sewage reference samples were prepared and processed in parallel with the above community sewage samples to assess the batch effects in the whole process. The ARG compositions of these reference samples were highly consistent, indicating negligible batch effects in comparison of sample metagenomic profiles obtained in different batches (Figure S1a,b).

### 2.2 Abundance, Diversity, and Composition of the Community Sewage Resistome

**FIGURE 1:**
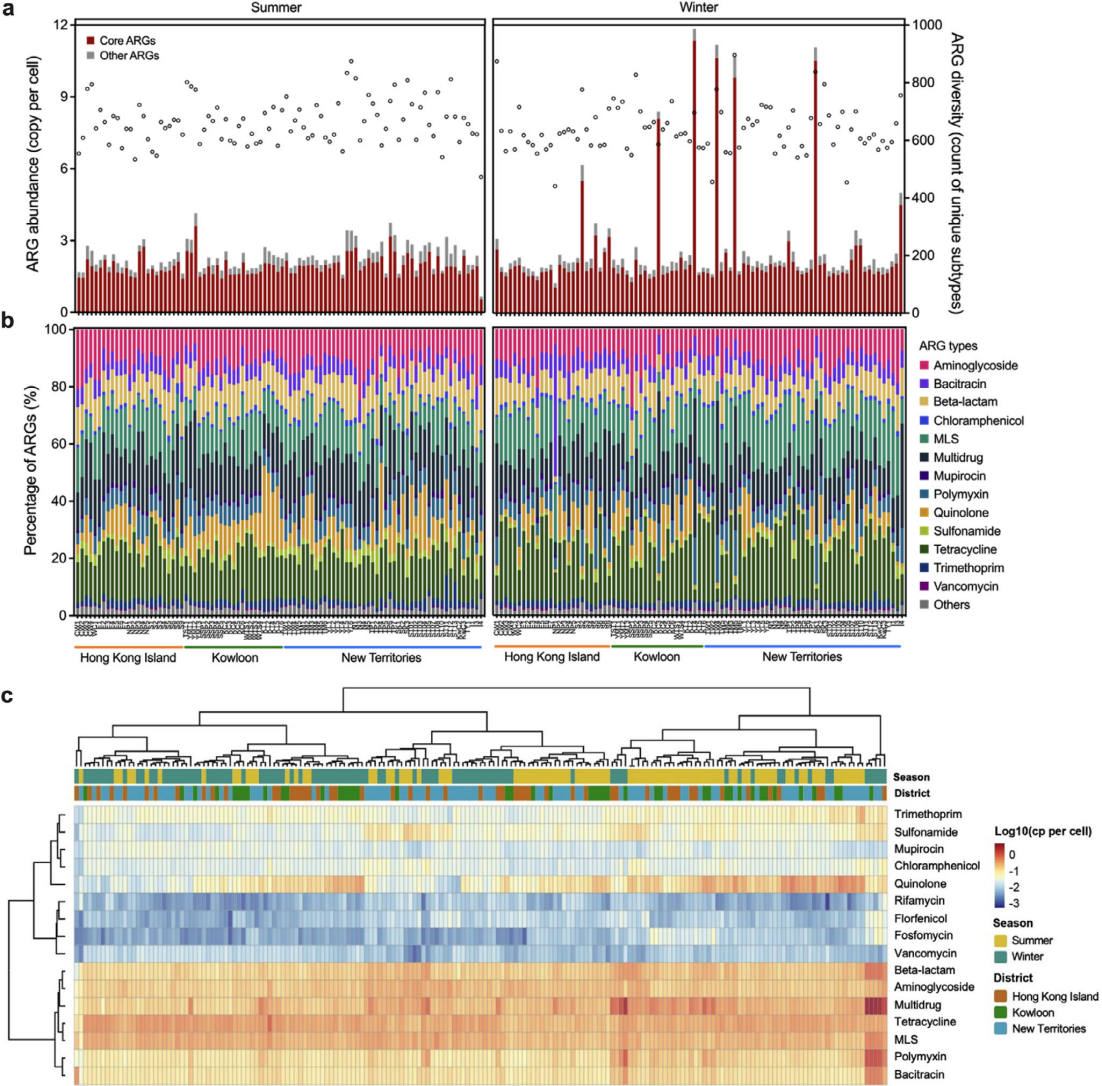
Overview of antibiotic resistome in Hong Kong community sewage. (a) Abundance and diversity of antibiotic resistance genes (ARGs) in Hong Kong community sewage (*n* = 185). Bars represented the ARG abundance in copies per cell, with the abundance of core resistome denoted in red. Circles represented the number of unique ARG subtypes detected in each sample. Sampling sites were arranged by district. (b) Percentages of ARG types in the community sewage. MLS was short for macrolide‐lincosamide‐streptogramin. (c) Heatmap and hierarchical clustering showing the resistome profiles at ARG type level. ARG abundances were log_10_ transformed. The dendrogram was created based on Euclidean distance with ward. D2 clustering method.

As derived from short‐read data, a total of 2,143 ARGs subtypes belonging to 27 ARGs types were detected among the Hong Kong community sewage samples, with 441–896 ARGs subtypes detected in different individual sampling sites (Figure 1a). The total ARG abundance ranged from 0.7 to 4.2 cp/cell (copies per cell) and 1.3 to 11.9 cp/cell in the summer and winter seasons, respectively. The highest ARG abundance was found in sampling site KT4 with 11.9 cp/cell, and the lowest was in sampling site I4 with 0.7 cp/cell. Additionally, by using methods proposed by Yin et al. [28] based on mass conservation, the ARG concentration in sewage was estimated to be 2.9 × 10^7^–3.0 × 10^8^ cp/mL (copies per mL) in summer and 2.8 × 10^7^–1.5 × 10^9^ cp/mL in winter (Figure S2). A total of 181 ARG subtypes resistant to 19 widely used antibiotic categories were shared across all samples, constituting 60.5%–96.3% of the total ARG abundance and only 8.4% of diversity, demonstrating their much higher abundance compared to less frequently occurring ARGs. ARGs conferring resistance to tetracycline, multidrug (referring to broad‐spectrum efflux pumps that could expel multiple classes of antibiotics), and macrolide‐lincosamide‐streptogramin (MLS) were the top three most abundant ARG types across the community sewage samples (Figure 1b). The dominant ARG subtypes in community sewage were *bacA* (bacitracin), *erm*(B) (MLS), *ugd* (polymyxin), *tet*(O) (tetracycline), and *qnrS2* (quinolone) (Figure S3). Notably, some sampling sites showed relatively high ARG abundance in the winter season, and multidrug resistance genes accounted for more than 40% of the total ARG abundance in these samples. Sampling sites with hospitals covered in the sewershed catchment exhibited higher ARG diversity (e.g., TST1, YL3, and ST4), yet their ARG abundance was comparable to that of other sites (Figure S4a,b). While only a weak positive association was observed between hospital bed counts and ARG diversity (Figure S4c), the elevated diversity at hospital‐influenced sites confirmed hospitals as critical nodes of the antibiotic resistome, underscoring the need for targeted management strategies of hospital effluents. When looking at clinically important ARGs [29], different distribution patterns were observed, and certain sampling sites turned out to be hotspots for specific ARGs. For example, higher levels of *mcr* were detected in New Territories, especially sampling sites YL6 and ST8; sampling sites in North and Island exhibited higher abundances of *tet*(X); *bla*_NDM_ was detected with higher abundance in sampling sites CW1, ST3, KT4, and I1, which highlighted the necessity for regional‐tailored AMR interventions (Figure S5).

A clear seasonal pattern of resistome profile was observed for both ARG type and subtype levels, while clustering based on the district was not obvious (Figure 1c and Figure S6). Although no significant difference was observed in total ARG abundance and most of the ARG subtypes were shared between summer and winter seasons (81.6% of the total detected ARGs), the abundance of some ARG types varied considerably between seasons (e.g., MLS, quinolone, and tetracycline resistance genes) (Figure S7a,b). Also, the ARG diversity in summer was significantly higher than that in winter (*p*‐value < 0.01) (Figure S7c).

We further compared the resistome profiling results obtained from Nanopore data to those from Illumina data. Overall, a strong correlation (*r* > 0.80) was observed for the abundance of most ARG types obtained by the two platforms, as indicated by the Pearson correlation analysis (Figure S8a). Discrepancies were observed for several ARG types, such as multidrug and quinolone resistance genes, which might be due to the characteristics of the ARG sequences (nucleotide distribution, GC content, etc.), and/or the sequencing biases of the platforms. The derived percentages of each ARG type were highly comparable (*r* = 0.94, Figure S8b).

### 2.3 Community Sewage Better Reflected Human Gut Resistome Than WWTP Influent

**FIGURE 2:**
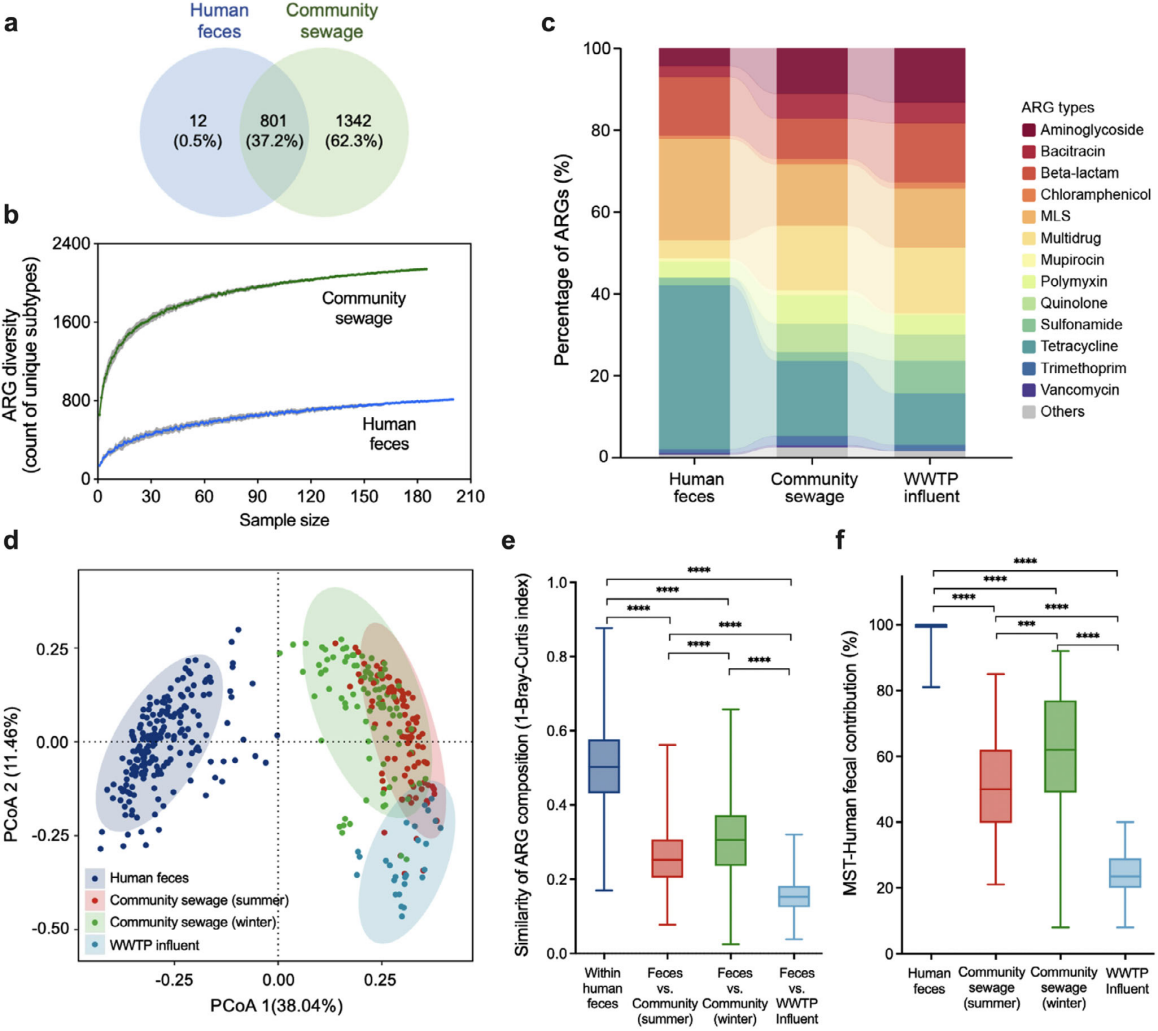
Comparison of ARG compositions in human feces and sewage metagenomes of Hong Kong. (a) The number of overlapping and unique ARG subtypes detected in human feces (*n* = 200) and community sewage (*n* = 185). (b) Rarefaction curves of ARG subtypes in human feces and community sewage of Hong Kong. We randomly subsampled 30 times for each sample type to count the average number of unique ARGs. (c) Stacked bar plots showing the mean percentages of ARG types in human feces, community sewage, and influent of wastewater treatment plants (WWTPs). (d) Principal coordinate analysis (PCoA) of overall resistome profiles based on the Bray–Curtis dissimilarity matrix at the gene level of ARGs. Colors denoted the sample types. (e) Similarity of community sewage and WWTP influent to human feces resistome. Half of the human fecal samples were randomly resampled to indicate resistome variation within human feces. (f) Contribution of human feces to community sewage and WWTP influent resistome as predicted by SourceTracker. Half of the human fecal samples were randomly resampled to assess the prediction performance. Mann–Whitney U tests were conducted for group comparison in (e) and (f), and *p*‐values less than 0.05 were considered statistically significant.

The human gut is considered the primary ARG reservoir and excretion route compared to other human microbiome sources. To determine to what extent the community sewage may resemble the human fecal resistome, which is generally used to represent the human gut resistome, we collected metagenomic datasets of human feces recruited from the Hong Kong public (see the Experimental Section). The total ARG abundances in Hong Kong human feces, community sewage, and WWTP influent were at similar levels, with an average abundance of 2.2, 2.5, and 1.8 cp/cell, respectively (Figure S9). However, the community sewage displayed higher resistome diversity than human feces, and almost all the fecal‐associated ARGs could be detected in the community sewage (98.5%), demonstrating sewage as an effective collective sample for population ARGs surveillance (Figure 2a,b). Human feces had a high proportion of tetracycline and MLS resistance genes, while the percentage of these genes decreased during the conveyance along sewers to WWTPs (Figure 2c). The three sample types (i.e., human feces, community sewage and WWTP influent) had distinct resistome structures, and clear clustering was observed through principal coordinate analysis (PCoA) based on the Bray–Curtis dissimilarity matrices (Figure 2d). Notably, the ARG composition of community sewage showed significantly higher similarity to the human feces than the downstream WWTP influent (*p*‐value < 0.05), and human feces contributed to an average of 50% (summer) and 60% (winter) of community sewage resistome, in contrast to only 23% of WWTP influent resistome (Figure 2e,f).

Similarly, the proportion of human fecal‐associated bacteria decreased during sewage conveyance (Figure S10a). For example, a significant decrease in abundance was observed for Bacteroidaceae, which was the predominant family in human feces, while the contribution of sewer‐specific families such as Arcobacteraceae, Moraxellaceae, and Rhodocyclaceae increased along the sewage system. The sample clustering pattern derived from PCoA based on the taxonomic composition was similar to that of ARG profiles, and Procrustes analysis revealed a significant correlation (M^2^ = 0.087, *p*‐value < 0.01) between the microbial communities and the ARG compositions (Figure S10b,c). Therefore, as sewage was conveyed to the WWTP, the shift of the resistome was closely related to the changes in bacterial communities.

### 2.4 *Escherichia coli* as an ARG Reservoir in Community Sewage

The Nanopore reads (median N50 = 7.3 kb, Table S1) were much longer than Illumina reads‐assembled contigs (median N50 = 2.9 kb, Table S2), which could help achieve better resolution for the genetic context analysis of ARGs. In total, 1,843,108 ARG‐carrying long reads were retrieved from the Nanopore data, encoding 1,452 unique ARG subtypes, while only 22,302 Illumina reads‐assembled contigs were found to carry ARGs. Given the current challenges in accurate plasmid host prediction, 1,336,831 (72.5% of total ARG‐carrying long reads) nonplasmid reads were kept for downstream taxonomy annotation, of which 97.0% could be assigned to the species level and retained for ARG host analysis (without short‐read correction).

**FIGURE 3:**
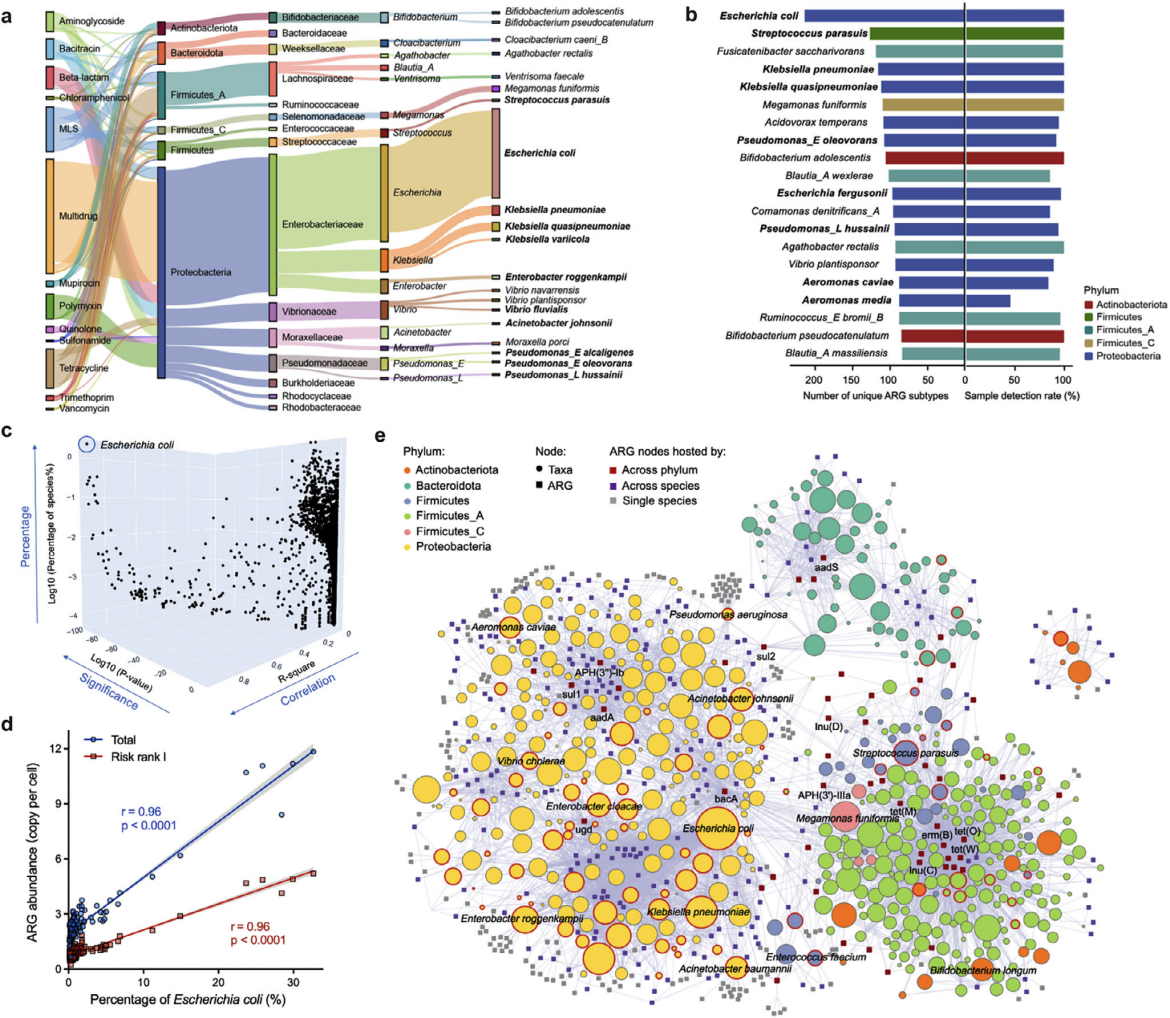
ARGs and their bacterial hosts in community sewage. (a) Sankey diagram illustrating the host range of ARGs at different taxonomy levels. The most abundant 20 species ranked by the ARG abundance were shown, with potential pathogenetic hosts highlighted in bold. (b) The ARG diversity and detection rate of bacterial hosts. The top 20 species that harbored the most diverse ARGs were shown. Different phyla were indicated in different colors. (c,d) The correlation between the percentage of all detected species (c) and *Escherichia coli* (d) with the ARG abundance across all samples. (e) Network analysis showing the co‐occurrence of ARGs and their assigned hosts at the species level. Nodes of species were denoted in different colors by their phylum, with pathogens labeled in red circles. ARG nodes shared by multispecies and multiphyla were highlighted in purple and red, respectively. Thickness of the edges denoted the number of reads supporting the ARG‐species links. Node size of species denoted their ARG abundance. The graph layout was manually adjusted for better visualization.

ARG‐carrying long reads were assigned to 15,642 bacterial species across 78 phyla, and 4.1% of the host species were identified as potential pathogenic bacteria, which were primarily affiliated with Proteobacteria. Notably, 42.5% of the chromosomal ARGs were found to be hosted by these pathogenic bacteria, demonstrating pathogens as major ARG carriers. A few ARG types tend to be carried by specific phyla (Figure 3a). For example, Proteobacteria was the predominant host phylum, contributing to more than 80% of the abundances of beta‐lactam, multidrug, polymyxin, and quinolone resistance genes. In contrast, tetracycline, trimethoprim, and vancomycin resistance genes were preferentially hosted by Firmicutes_A, which was the second‐largest contributor of ARGs. Remarkably, *Escherichia coli* was the host for 27.9% of all chromosomal ARGs and up to 214 ARG subtypes (belonging to 20 ARG types), which was much higher than other host species (Figure 3a,b). ESKAPE pathogens, such as *Klebsiella pneumoniae*, *Acinetobacter baumannii*, and *Pseudomonas aeruginosa*, were also found to carry a high diversity of ARGs, which may challenge their infectious treatment by antibiotics (Figure S11). Interestingly, we found that the percentage of *E. coli* showed the highest degree of correlation with both total and risk Rank I [23] ARG abundance among all detected species (Figure 3c,d). Together with its high prevalence across samples, *E. coli* could be a potential indicator bacterium for sewage‐based AMR surveillance, which was consistent with previous studies [30, 31].

The ARG‐host cooccurrence network graph was further constructed to investigate the ARG‐sharing status between host species. We found that some ARGs were limited to specific hosts. However, a large portion of other ARGs were shared between taxa, among which *erm*(B) (MLS), *tet*(W) (tetracycline), and *lnu*(C) (MLS) were the ARGs associated with the highest number of host species (Figure 3e). Interestingly, only a few ARGs were hosted by multiple phyla. A phylum‐level clustering was observed, indicating that phylogenetic barriers constrained the dissemination of the majority of ARGs, which was in accordance with previous studies [32, 33, 34]. Again, *E. coli*, as well as the ESKAPE pathogens, were found to share a large number of ARGs with other species, indicating their significant roles in ARG transmission.

### 2.5 Mobility Potential of ARGs in Community Sewage

**FIGURE 4:**
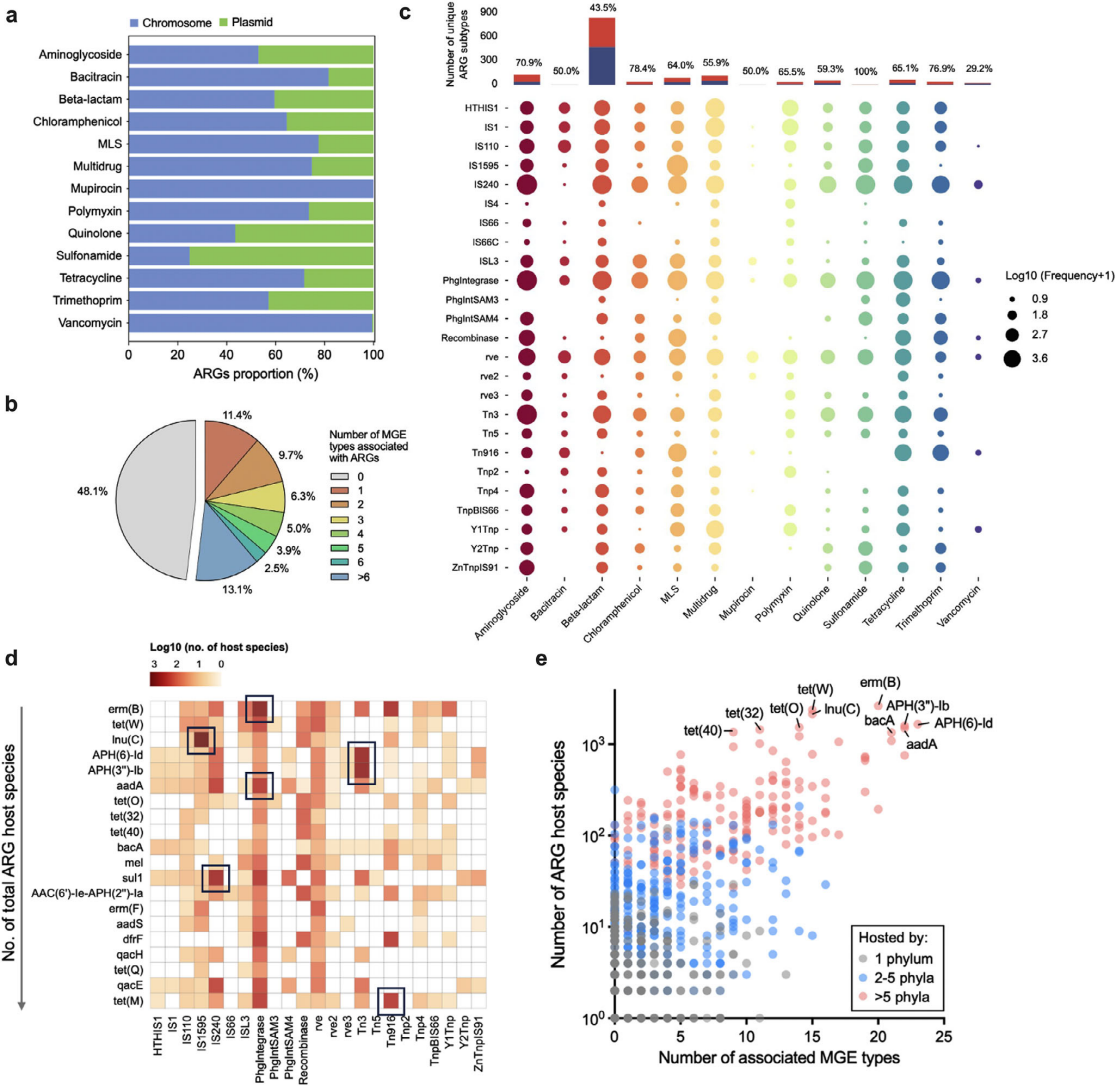
Analysis of ARG mobility potential. (a) The distribution of ARGs on plasmids (green) and chromosomes (blue) by ARG types. (b) Pie chart illustrating the proportions of ARGs associated with one or more types of adjacent MGEs within 5 kb flanking regions (among the 1,423 detected ARGs). (c) The association of ARGs with adjacent MGEs. Circle size denoted the ARG abundance in each ARG type located in close proximity to the corresponding MGE types. Top bar chart showing the number of unique ARG subtypes found to be associated (red) or not associated (blue) with MGEs. Labeled numbers were the percentage of MGE‐associated ARGs in each ARG type. (d) Heatmap showing the host range of ARGs associated with specific MGE types. Only the top 20 ARGs with the broadest phylogenetic reach were presented. Black boxes indicated the ARG‐MGE pairs that were detected across all samples. (e) The relationship between the host ranges of ARGs and the number of MGE types they were associated with. Each point represented an individual ARG subtype. The top 10 ARGs with the most diverse host species were labeled.

The 506,277 ARG‐carrying plasmid reads were considered for further analysis of the mobility potential of ARGs. As expected, ARGs were more frequently detected on the plasmid sequences than on the chromosomes (*p*‐value < 0.01, Figure S12), confirming plasmids as major vectors of ARGs. Around 30.2% of ARG abundances belonging to 1,016 unique ARG subtypes were found to be located on plasmids, among which 82 ARG subtypes were only associated with plasmids, including *bla*_NDM_—a carbapenemase of clinical concern typically located on plasmids [35]. Different ARG types exhibited varying mobility potential, with sulfonamide‐ (75.2%), quinolone‐ (56.4%), aminoglycoside‐ (47.0%), and trimethoprim‐ (42.9%) resistance genes being more frequently carried by plasmids (Figure 4a and Table S3).

MGEs are capable of shuffling ARGs between bacterial cells or between chromosomes and plasmids/phages within the same cell, thus facilitating resistance transmission and maintenance [36]. Our results showed that MGEs was strongly correlated with ARGs in both abundance and diversity (Figure S13). We then searched for ARGs that were in close proximity to the putative MGEs (5 kb upstream and downstream) to identify potentially mobile ARGs. A total of 753 ARG subtypes were found to have MGEs in the flanking regions, of which 588 (78%) were associated with more than one type of MGEs (Figure 4b). Each ARG type was apt to have different MGEs nearby, with aminoglycoside and beta‐lactam resistance genes associated with the highest number of unique MGEs, and these two ARG types constituted a large proportion of the potentially mobile ARGs (Figure 4c). Of note, IS240 was the MGEs occurring in proximity with most diverse ARGs (473 subtypes), including clinically important *bla*_CTX‐M_, *bla*_KPC_, *mcr*, and *tet*(X). The ARG‐MGE combination of *aadA*‐PhgIntegrase, *APH(3'')‐Ib*‐Tn3, *APH(6)‐Id*‐Tn3, *erm*(B)‐PhgIntegrase, *lnu*(C)‐IS1595, *sul1*‐IS240, and *tet*(M)‐Tn916 were detected across all samples. These prevalent ARG‐MGE pairs were observed to have a wide host range, indicating the pivotal role of these MGEs in mediating the transfer of ARGs (Figure 4d). Additionally, ARGs associated with more types of MGEs in the surrounding regions demonstrated higher dissemination potential and were likely to have broader phylogenetic reach (Figure 4e), which was consistent with previous studies that the spread of ARGs was constrained by their associated MGEs [33]. However, some ARGs with multiple adjacent MGEs still exhibited a limited number of host species, which might be due to each ARG's distinct dissemination patterns and functional constraints.

### 2.6 Transmission Mode and Status of ARGs in Hong Kong Communities

Horizontal and vertical gene transfer are two major mechanisms for ARG dissemination [37]. To assess potential health risk of ARGs, it is of great importance to understand the transmission mode and status of ARGs, especially those clinically relevant ones. As the relatively high error rate of Nanopore raw reads precluded mutation detection, the Nanopore reads were then assembled and polished with Illumina reads to improve sequence quality for better ARG homology analysis and genetic context retrieval.

Overall, a total of 6,261,029 contigs were yielded from all samples with a median N50 length of 48.7 kb (Table S2), of which 40,454 were identified as ARG‐carrying contigs. Only those with at least 5 kb flanking regions to the annotated ARGs were then retained for the following analysis. Homology analysis, host association, and genetic organization of ARG variants with clinical relevance were carried out to deduce their local spread and future dissemination potential, assuming that highly similar ARGs detected in multiple host species with neighboring MGEs were caused by HGT [38]. As expected, ARGs displayed diverse transmission patterns and could be categorized into four major types.

**FIGURE 5:**
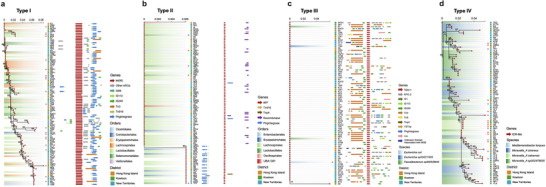
Transmission patterns of clinically relevant ARGs with varying risk levels revealed by ARGs’ homology and their genetic contexts, as represented by (a) *tet*(M), (b) *dfrF*, (c) *bla*_TEM‐1_, and (d) *ICR‐Mo*. For each ARG subtype, a phylogenetic tree was constructed using multiple sequence alignment of all detected variants with at least 5 kb flanking regions. Topological confidence is displayed as numbers adjacent to the nodes. Assigned bacterial hosts of ARGs were presented in gradient colored shade, with potential pathogenetic hosts depicted in red asterisks, and no colors were applied for ARGs located on plasmid contigs. Sampling sites and corresponding districts of the detected ARGs were indicated on the right side of the phylogenetic trees. The gene arrangement of each variant was plotted as colored arrows, with targeted ARGs colored in red, other ARGs colored in grey, and associated flanking MGEs (5 kb upstream and downstream of the ARGs) colored according to their types.

Type I ARGs showed characteristics of wide host range and high sequence heterogeneity, as exemplified by *tet*(M) (tetracycline). *tet*(M) was detected in 37 bacterial species across four phyla (Figure 5a), exhibited high sequence divergence from the orthologs, and mostly associated with MGEs Tn916 and PhgIntegrase. This suggested that this ARG might have disseminated among diverse hosts in local communities and develop high sequence divergence, and it still possessed potential for future dissemination given its strong associations with MGEs.

Type II ARGs were characterized by a wide host range but high sequence conservation, as exemplified by *dfrF* (trimethoprim). *dfrF* was carried by 52 species across three phyla, mostly affiliated with Lachnospirales, and occasionally found on plasmids, but its sequences were highly similar, with only two clades of close phylogenetic distances (Figure 5b). The genetic patterns associated with MGEs were well‐conserved, with the first clade located upstream of MGE Recombinase and the second clade in proximity to MGE Tn916 and PhgIntegrase, which were speculated to be responsible for the mobilization of *dfrF*. The high dissemination potential of these ARGs made them more likely to transfer from commensal gut bacteria, like Lachnospirales, to human pathogens and endanger public health.

Type III ARGs were mostly plasmid‐associated, which showed widespread occurrence across different local regions, as exemplified by *bla*_TEM‐1_ (beta‐lactam) (Figure 5c). The sequences of *bla*_TEM‐1_ were highly similar, while its genetic structures varied considerably, involved with different types of MGEs, and sometimes cooccurred with ARGs that confer resistance to other antibiotics (e.g., aminoglycosides and trimethoprim). Although the exact host range of plasmids was not investigated in the present study, these plasmid‐associated ARGs showed potential to transfer across bacterial hosts and might accelerate the further dissemination of antibiotic resistance.

Type IV ARGs showed narrow host ranges and exhibited relatively low mobility potential, with no MGEs observed in their flanking regions or carried by plasmids, as exemplified by *ICR‐Mo* (polymyxin). *ICR‐Mo* was found to be exclusively chromosomally encoded and evolved within *Moraxella_A* species (Figure 5d), indicating that its transmission might primarily rely on the proliferation of bacterial hosts to their offspring. Therefore, the group of ARGs might have a low potential health risk as it cannot jump to human pathogens easily.

## 3 Discussion

### 3.1 Upstream Sampling Strategies Show Superiority in Reflecting Human‐Sourced Resistome

Due to the transmission complexity of AMR, enhanced AMR surveillance is urgently needed to understand to what extent and in which way AMR spreads. Metagenomics‐based sewage surveillance has been considered a promising tool to mirror the overall AMR profiles of the communities in the catchment areas, including carriage among both patients and healthy populations [7]. However, in addition to human waste, sewage contains microorganisms from multiple sources. Unlike surveillance of infectious disease agents that were mainly derived from human bodies, sewage resistome could have human and environmental origins, and to what extent sewage could mirror population‐level AMR burden needs to be determined. The spatial resolution for sewage surveillance programs varied from building levels to WWTP levels, with sewer manholes and WWTP inlets being the typical sampling sites. By comparing with local human feces metagenomic data, we found that the community sewage, geographically very close to human hosts, could better represent the gut resistome of the human population than WWTP influent, although its resistome was still distinct from that of human feces. Interestingly, the community sewage comprised almost all ARGs detected in the human fecal dataset, highlighting its value as composite samples to collectively monitor ARGs that circulated within local populations.

The resistome compositions shifted as human feces passed through the sewage system, and the community sewage resistome has high similarity to the human feces resistome compared to that of the WWTP influent. Our analysis showed that this was closely associated with bacterial community change, which might be due to the attenuation of obligate anaerobic human fecal bacteria as they entered the aerobic environment [39], as well as the increase of resident bacteria from biofilm shedding and turbulence‐induced sediment resuspension [40]. The nonhuman‐sourced inputs would presumably interfere with the sewage surveillance of ARGs, and more upstream sampling strategies could help diminish this kind of stochastic noise. A recent study also demonstrated that sampling sites closer to human source could better reflect human‐derived microorganisms [22]. These findings could be helpful for better interpretation of the sewage metagenomic data for epidemiological purposes in future efforts.

It should be noted that Hong Kong adopted a separate sewer system without rainwater inputs, and the total traveling distance of sewage from residential buildings to WWTPs was relatively short, which limited opportunities for environmental bacteria introduction. Despite this, a significant human fecal “signal dilution” was observed from upstream sampling sites to WWTP influent. When extrapolating this finding to areas with combined sewer systems or longer sewage transportation time, the disparity could be even more pronounced. For example, in studies conducted in USA [41] and Canada [42], human‐derived microorganisms were reported to represent only 10%–15% and 7% of the microbial communities of the WWTP influent. While there is a trade‐off between spatial resolution, local deployability, and cost‐effectiveness, the optimal location of sampling sites should be carefully designed and selected, which might be supported by algorithms [43].

### 3.2 Short‐Term Bloom of Specific Microorganisms Should Receive Further Attention

We found diverse and abundant ARGs belonging to almost all types of antibiotics in the community sewage. ARGs conferring resistance to tetracycline, multidrug, and MLS constituted the highest relative abundance, with *bacA*, *erm*(B), *ugd*, *tet*(O), and *qnrS2* being the dominant subtypes. This could stem from multiple interconnected factors related to antibiotic usage, environmental selective pressure, genetic mobility, and resistance mechanisms. It should be noted that *bacA* was observed to encode undecaprenyl pyrophosphate phosphatase in *E. coli* (involved in bacterial cell wall biosynthesis) [44], and its overexpression has been shown to result in bacitracin resistance [45]. Consequently, the observed high *bacA* abundance in our samples should be interpreted cautiously since metagenomics alone cannot confirm its phenotypic resistance level. Future studies employing transcriptomics and phenotypic assays are essential to elucidate the functional contribution of *bacA* and similar genes to the resistome. The prevalence of most ARG subtypes between summer and winter seasons suggested the consistent sources and stable microbial reservoirs of community sewage. The core resistome comprised only 181 ARG subtypes while contributing to a large portion of the total resistome abundance, underscoring their significant roles in shaping the resistome profile and the necessity for examining these persistent and abundant ARGs. Similar results regarding the core resistome of urban sewage [46] and activated sludge [47] have been previously reported. Seasonal variation driven by significant abundance changes of specific ARGs was observed, potentially linked to differences in antibiotic usage and sewage temperature. While local prescription data were unavailable, increased antibiotic consumption during winter seasons—as documented in other Chinese cities—offers a plausible explanation for this pattern [48, 49]. Sewage temperature, inferred from average ambient temperature during summer (29 °C) and winter (18 °C) sampling periods, likely further influenced the resident sewer bacterial communities and associated ARG profiles [41]. Moving forward, multisector collaboration will be essential to build integrated datasets that could better resolve the seasonal dynamics of ARGs. No distinct clustering based on the district was observed, possibly due to the relatively small geographical range of this study and high social interaction intensity within these densely populated areas.

Remarkably, several sampling sites showed relatively high ARG abundance in the winter season, which was most likely caused by *E. coli*, as we found the predominance of *E. coli* in these samples. Such short‐term bloom of *E. coli* might be caused by norovirus‐driven gastroenteritis outbreaks in the population (which occur more commonly in winter [50]), and the infection was observed to disrupt the gut microbiota with a significant increase in *E. coli* [51]. The clinical surveillance data based on fecal sample testing revealed a clear peak in Norovirus detections in January 2022 (Figure S14). The alignment of this peak with the winter sampling period further substantiated the hypothesis. However, the potential influence of other contributing factors on this phenomenon cannot be ruled out, such as resistant bacteria enrichment due to seasonal increases in antibiotic prescriptions [48], prolonged bacterial survival due to lower sewage temperatures [52], and in‐sewer bacterial growth due to reduced sewage flow rate [53]. The representativeness of these samples in this study for ARGs in the population needs further investigation, and more longitudinal surveillance is suggested to provide a comprehensive picture. Also, although sampling at sewer manholes, the population mixing and infrastructure homogenization could still dilute localized signals, and more targeted upstream sampling at single‐function infrastructures (e.g., residential buildings, schools, and hospitals) could better resolve resistome in the community sewage.

### 3.3 Long‐Read Sequencing Could Extensively Uncover the Host Range and Mobility of ARGs

The considerably long reads generated by Oxford Nanopore sequencing demonstrated unparalleled advantages for ARG contextual analysis, as they may span the repetitive regions commonly flanking ARGs that are difficult to assemble from the highly fragmented short‐read data. Advancing AMR management requires comprehensive databases that contain not only the occurrence of ARGs but also their host ranges to help determine the risk of ARGs to human health and develop targeted mitigation strategies to mitigate their spread [54]. We found that the majority of ARGs in community sewage originated from members of Enterobacteriaceae, many of which were closely associated with clinical infections, such as *Escherichia, Klebsiella*, and *Enterobacter* [55]. ARGs conferring resistance to last‐resort antibiotics, such as carbapenemase genes and colistin resistance genes, were detected and occasionally carried by human pathogens, which should receive further public health attention.

It should be noted that the host‐tracking of ARGs could be biased by the taxonomic classification tool and the reference database available. To achieve reliable ARG host assignment, only sequences with ≥ 1 kb flanking regions were used in this study. However, there could still be some misclassifications between closely related taxa, and species not represented in the current database remain unidentifiable. Also, metagenomes were unable to provide high‐confidence host assignment information for plasmid‐born ARGs, especially for those carried by broad‐host‐range plasmids. Thus, the plasmid sequences were not included in the host analysis in this study. The exact host range of those plasmid‐associated ARGs could be investigated through advanced genomic analysis, such as DNA methylation profiling. In addition, other culture‐independent techniques, such as single‐cell sequencing, genomic cross‐linking, and fusion PCR (epicPCR), could also help link ARGs with their hosts in complex communities [54].

The mobility of ARGs is suggestive of their transmission potential and essential for assessing their health risk. Taking advantage of long‐read sequencing, we obtained a comprehensive picture of the mobile resistome in Hong Kong community sewage. We found that a larger proportion of ARGs were located on chromosomes, and the transfer potential of ARGs encoding resistance to different types of antibiotics varied significantly, with sulfonamide resistance genes exhibiting the highest mobility due to their close association with class 1 integrons [56]. To provide direct evidence, a representative Nanopore long read harboring the *intI1*‐*qacEΔ1*‐*sul1* cassette array in our dataset has been deposited in GenBank (BioProject PRJNA1207805). No significant seasonal difference was observed regarding the genetic location of ARGs, with 29.5% and 30.7% of ARG abundances located on plasmids in summer and winter, respectively. It should be noted that metagenomic data might partly underestimate the plasmid content, and plasmid enrichment processes could help better reveal the plasmid resistome of the samples [57].

By searching for MGEs in the flanking regions of ARGs, we further identified ARGs that were possibly mobilized by MGEs. Many ARG‐MGE combinations were detected in our data, displaying varying phylogenetic reach, many of which were sometimes carried by human pathogens, thus posing high risks to public health. IS240 family stood out as the most noteworthy, associated with the highest number of unique ARGs, coinciding with previous findings [33]. Insertion sequences (ISs) are known to play important evolutionary roles in genome plasticity [58]. Although they could not directly assist the intercellular movement, the interaction between conjugative plasmids and ISs has been demonstrated as a significant mechanism for the horizontal transfer of ARGs [38]. Interestingly, we found phage integrase frequently co‐occurred with a wide range of ARGs, similar to results in a previous study [59]. While there is still controversy over the contribution of phages to the resistome [60], the role of phages in spreading ARGs should be further explored.

### 3.4 Resolving ARG Variants and Their Genetic Contexts May Enhance the Existing ARG Risk Assessment Framework

Despite the sequencing accuracy of Nanopore long reads generated using Q20+ chemistry being largely improved for better gene identification (mean read quality of 20.3, Table S1), potential basecalling errors still hindered the detection of actual sequence variants and homology analysis. For this reason, we used short‐read‐polished long‐read assemblies to analyze the individual ARGs as well as their genetic contexts.

Based on the assumption that highly similar ARG sequences detected across taxa were caused by gene mobilization [38], the sequence diversity of ARGs, together with the arrangement of their neighboring genes, were used to deduce the evolution of ARGs. We found diverse ARG transmission patterns in Hong Kong community sewage, each with different degrees of potential risk: (1) Type I ARGs exhibited higher sequence divergence and more complicated genomic background, which might be shaped by their widespread transmission and diverse evolutionary processes, indicating their existing high risk to local populations. These ARGs typically conferred resistance to antibiotics with early and wide clinical usage. Continuous tourism and immigration that introduced exogenous ARG variants could also contribute to this. (2) Type II ARGs displayed well‐conserved sequences and flanking MGEs across multiple bacterial species and plasmids, implying their relatively short time since first emergence in local communities and frequent HGT events. Intensive monitoring and effective intervention strategies will be beneficial to manage their potential spread in the future. (3) Type III were those plasmid‐born ARGs that showed highly identical sequences with varying genetic context, which suggested their potential transfer and continuous reorganization among plasmid sequences. These ARGs should receive further attention, and studies should be conducted in the future to determine their occurrence among bacteria. (4) Type IV ARGs showed narrow host ranges and were rarely flanked by MGEs, which were supposed to spread mainly through vertical gene transfer (VGT). These ARGs demonstrated relatively low mobility potential for future dissemination and would pose a lower risk to public health.

We hope to use this as a demonstration to provide insights for enhancing the current ARG risk assessment framework from an evolutionary perspective. However, it should be noted that our evolutionary inference relied on the assumption of uniform mutation rates. Since mutation rates can vary across species in reality [61], this simplification may affect divergence time estimation. Furthermore, the varying fitness cost among mutants [62] and the occurrence of compensatory mutations [63] may also confound the interpretation. For instance, in addition to HGT events, sequence conservation of an ARG could also arise from purifying selection due to functional constraints [64] or from compensatory mutations that mitigate fitness costs without altering the ARG sequences [65]. Therefore, while our framework offered a practical tool for prioritizing ARGs with different observed dissemination characteristics, future studies incorporating evolutionary rate analyses and experimental validation will be essential to delineate their precise evolutionary history.

Notably, relying solely on ARG sequence similarity comparisons may lead to overestimating gene transfer events. Incorporating an analysis of the genomic background could help achieve more conservative and accurate conclusions. Besides, this could facilitate our understanding of the transmission mechanism of specific ARGs regarding their transfer modules. For example, *lnu*(C) (MLS) was found to be almost exclusively flanked by IS1595, an ISXO2‐like transposase domain, across distinct bacterial species, and this gene arrangement has been previously reported in other studies [66]. Interestingly, we found varying completeness of specific MGEs that flanked a certain ARG, which might be caused by their dynamic evolution when ARGs stabilized in new hosts [38]. Also, it is important to keep in mind that some neighboring MGEs might not be involved in ARG mobilization (e.g., IS5 was reported to be possibly responsible for modulating the expression of *tet*(W) rather than mobilization [67]), and their actual mobility needs further in vitro experimental validation.

The analysis of transmission patterns underscored the importance of incorporating long‐read sequencing into AMR metagenomic surveillance to better uncover the modularity of ARGs, track their phylogenetic prevalence, and predict their future dissemination. As this study only focused on Hong Kong community sewage, we did not observe distinct geographical clustering for transmission, and the generalizability of the transmission pattern to other regions remains uncertain. Further research is needed to explore the global dissemination patterns of ARGs.

## 4 Conclusions

As one of the greatest challenges of the 21st century, AMR shows a significant impact on both public health and the economy, highlighting the urgent need for enhanced AMR surveillance. In this study, we reported the first work combining both long‐read and short‐read sequencing technologies for large‐scale AMR sewage surveillance to comprehensively characterize the antibiotic resistome of community sewage across Hong Kong. We observed significant seasonal patterns in the high‐resolution resistome profiles as driven by variation of specific ARG types, while no distinct spatial pattern was found. Notably, community sewage was demonstrated to be a superior proxy to human gut resistome compared to WWTP influent, suggesting it as a robust sentinel for monitoring population‐level AMR burdens. The documentation of a substantial human‐associated‐ARG reduction during sewage conveyance provides insights for antibiotic resistance management and caution for WWTP influent‐based antibiotic resistance surveillance. The integration of long reads significantly advanced the resolution of ARG host tracking and genetic context profiling, elucidating previously underexplored ARG dynamics. By further combining with the phylogenetic diversity of ARGs, this study identified four antimicrobial resistance transmission patterns. Among the transmission patterns, some ARGs exhibited long‐term local transmission, some demonstrated potentially frequent HGT, while others primarily propagated through VGT. These could provide new insights into the spread of ARGs and their risks to public health.

We believe that the findings of this study will guide future efforts toward highly‐resolved metagenomic‐based longitudinal AMR sewage surveillance to track AMR trends and transmission patterns, thereby informing more efficient AMR management strategies. As demonstrated here by its application on a megacity scale, the large‐scale use of long‐read sequencing, complemented with short‐read sequencing, could significantly enhance global AMR surveillance efforts. The approach established in this study offers a baseline framework to support advancing sewage metagenomic surveillance in the future.

## 5 Experimental Section

### 5.1 Sewage Sample Collection and Pretreatment

The 3‐h composite community sewage samples were collected in the morning peak (8–11 am, 15‐min interval) from manholes at 95 stationary sites (upstream in the sewage collection system compared to WWTP inlets) across all 18 districts in Hong Kong using the autosamplers, with approximately 1 L per sample. These sampling sites had population sizes ranging from 10,000 to 264,000 people, covering 68.2% of the Hong Kong citizens in total. In total, 190 samples were taken in two separate sampling campaigns in May 2021 and January 2022 to account for seasonal differences, during which there was no significant COVID‐19 case surge in Hong Kong. All the samples were delivered to the laboratory and underwent immediate processing. For each sample, pellets were obtained from 40 mL of the sample after centrifuging at 4,750 × g for 30 min and stored at −80°C until DNA extraction.

### 5.2 DNA Extraction and Purification

DNA extraction for the sewage samples was conducted using the DNeasy PowerSoil Pro Kits (Qiagen, Germany) on the QIAcube Connect Extraction System (Qiagen, Germany) with a final elution volume of 50 µL. All DNA samples were purified using AMPure XP beads (Beckman Coulter) at 1:1 bead: sample ratios. DNA concentrations and purity were measured using a Qubit Fluorometer (ThermoFisher Scientific, USA) and NanoDrop Microvolume Spectrophotometers (ThermoFisher Scientific, USA), respectively. High‐quality DNA samples (OD 260/280 ratios of 1.8–2.0 and OD 260/230 ratios of 2.0–2.2) were used for Nanopore and Illumina sequencing.

### 5.3 Illumina Sequencing and Data QC

The construction and sequencing of shotgun libraries were conducted for samples with sufficient DNA mass on the Illumina NovaSeq6000 platform using the PE150 strategy by the Shanghai Majorbio Bio‐pharm Technology Co., Ltd, and the average depth was ∼10 Gb (Table S1). The metagenomic reads were filtered using fastp v0.23.4 [68] with default parameters. Human sequences were identified by mapping reads to the human reference genome (T2T‐CHM13 v2.0) [69] using bowtie2 v2.5.4 [70]. Paired reads were removed if either read was mapped to the human genome.

### 5.4 Nanopore Sequencing and Data QC

Nanopore sequencing libraries were prepared using the Native Barcoding Kit 24 V14 (SQK‐NBD114.24) following the manufacturer's instructions. Barcoded samples were pooled together, and the final libraries were sequenced on the Oxford Nanopore PromethION R10.4.1 flow cells (FLO‐PRO114M) for at least 72 h. Community sewage samples with sufficient DNA mass were subjected to Nanopore sequencing, and a total of 32 PromethION flow cells were used (Table S1). The raw Nanopore fast5 data were basecalled using Guppy v6.5.7 with the SUP model to return fastq files. The demultiplexed Nanopore reads were filtered using NanoFilt v2.8.0 (https://github.com/wdecoster/nanofilt) to remove reads shorter than 1000 bp, and low‐quality reads with average quality scores of less than 10. Human sequences were removed by mapping reads to the human reference genome using minimap2 v2.26 [71].

### 5.5 Process Control

As the batch effect is one of the major concerns for large‐scale metagenomic analysis, we prepared sewage reference samples (well‐homogenized mixtures of influent samples collected from 11 WWTPs) following the protocol of Yang et al. [72] Briefly, 500 mL of each sample was centrifuged at 4,750 × *g* for 30 min, and the pellets were resuspended using phosphate‐buffered saline (PBS) to 20 mL. All samples from 11 WWTPs were mixed, then transported into a flask and orbitally shaken at 180 rpm for 2 h with silica beads. The homogenized sample was magnetically stirred and aliquoted into equal portions of 2 mL each. Pellets of aliquots were collected after centrifuging at 20,000 × *g* for 3 min and stored at ‐80°C till further use. We processed one reference sample in parallel to the sewage samples in each batch to assess the quality of our DNA extraction, library preparation, and sequencing procedures. These sewage reference samples with similar levels of microbial composition complexity as community sewage allow for a holistic evaluation of batch effects on sample profiling as a part of QA/QC (quality assurance and quality control) to ensure reliable metagenomic analysis.

### 5.6 Collection of Datasets of Human Feces and WWTPs Influent

Human feces metagenomic data of the Hong Kong population cohort (*n* = 200) were retrieved from the NCBI SRA database (accession PRJNA557323). There were 116 females to 84 males, self‐reported as healthy. Their median age at the time of sample collection was 53 years (SD 16.4 years), and their median body mass index was 22.7 kg/m^2^ (SD 3.2). Influent metagenomic data of 14 Hong Kong WWTPs (*n* = 28) were obtained from our another project. Fastq reads went through the same QC workflow as for the community sewage data. To estimate the proportions of human feces contributed to the community sewage resistome and WWTP influent resistome, modeling of microbial source tracking (MST) was conducted using SourceTracker v1.0.1 with default settings [73] based on ARG compositions at the gene level.

### 5.7 Annotation of ARGs and MGEs

For Illumina data, ARGs were detected and quantified by aligning reads using the ARGs‐OAP pipeline v3.2.2 with default settings [74], where the abundance of ARGs was normalized by cell numbers. Following the same procedure, MGEs in the samples were quantified by replacing the reference database with a comprehensive MGE database [33] in the ARGs‐OAP v3.2.2, with the unit of copies of MGEs per cell.

For Nanopore data, ARGs were identified by aligning reads against the SARG database v3.2.2 using the BLASTX function of DIAMOND v2.1.8 [75] at a maximum e‐value of 1e‐20, a minimum identity of 80%, and a minimum subject coverage of 80%. The ARG abundance in the unit of copies of ARGs per cell was calculated using the following equation [76]

(1) $$\mathrm{Abundanc}e_{\mathrm{ARG}}=\frac{\sum_{i=1}^{n}R_{i}}{L\times N}$$

where *n* represents the number of reads carrying the specific ARG; *R_i_* represents the alignment length of the specific ARG on read_i_; *L* represents the length of the specific ARG reference sequence; *N* represents the genome copy number in the dataset derived by Melon v0.1.0 [77].

Identification of MGEs located on the ARG‐carrying reads was performed using minimap2 v2.26^71^ against the MGE database mentioned above with a minimum identity of 80% and a minimum subject coverage of 80%. The ARG‐MGE co‐occurrence patterns were studied based on ARGs and their adjacent MGEs within 5 kb upstream and downstream regions [78].

### 5.8 Plasmid Classification

Nanopore reads with at least 1 kb additional length besides the ARG‐mapped regions were used for downstream analysis. To identify the ARG‐carrying reads with potential plasmid origin, the following three methods were used: (1) geNomad v1.7.4 with default parameters [79], (2) PLASMe v1.1 with default parameters [80], (3) reads aligned to PLSDB 2023_11_03_v2 [81] with minimum identity 80% and minimum query coverage 80% using minimap2 v2.26 [71]. Reads annotated as plasmid sequences by at least two of the three methods were determined as plasmid origin.

### 5.9 Taxonomy Classification and ARG Host Identification

The microbial compositions for Illumina data were classified using Kraken2 v2.1.3 [82] with a custom database generated from GTDB (release 207) [83], and the percentages of taxa in each sample were further estimated by Bracken v2.9 [84]. Taxa with percentages lower than 0.01% were removed from the profile to avoid false positive results. Identification of ARG hosts for Nanopore reads was also performed using Kraken2 v2.1.3 with the same above database. Only nonplasmid reads assigned to the species level were used for downstream analysis. Pathogenic hosts were identified by comparing the species‐level taxonomic results to the gcPathogen database [85] (modified based on the GTDB taxonomic system).

### 5.10 Homology and Phylogenetic Analysis of ARGs and Genetic Context

To further look into the ARG variant sequences and their genetic contexts, the Nanopore reads were de novo assembled using Flye v2.9.2 [86] with the mode “—meta,” followed by three cycles of polishing using Illumina reads with Pilon v1.24 [87]. The open reading frames (ORFs) of the corrected contigs were predicted using Prodigal v2.6.3 [88] with the “‐p meta” option. The ARGs‐like ORFs were identified using the BLASTP function of DIAMOND v2.1.8 against the SARG database v3.2.2 at *e*‐value ≤ 1e‐20, identity ≥ 80%, and subject coverage ≥ 80%. The 5 kb upstream and downstream regions of observed ARGs were extracted using BEDTools v2.31.1 [89], and the nearby MGEs were determined using BLASTN [90] against the above‐mentioned MGE database at e‐value ≤ 1e‐10 and identity ≥ 80%, where coverage was not filtered to include MGEs that might be truncated during evolution [38]. Contigs with shorter than 5 kb upstream and downstream flanks were excluded from the analysis. The sequences of ARGs were subjected to multiple alignment using Clustal Omega v1.2.4 [91], and the maximum likelihood tree was then constructed using IQ‐TREE2 v2.3.4 [92] with the following parameter settings: “‐m MFP ‐B 1000 –alrt 1000.” The hosts of these ARGs were subsequently analyzed as described above.

### 5.11 Statistical Analysis

Data were analyzed by Mann–Whitney U tests and Wilcoxon signed rank tests for data comparison, and a *p*‐value of less than 0.05 was considered statistically significant. Geographical location and resistome distribution of different sampling sites were visualized using QGIS v3.34. Data figures were created using GraphPad Prism v9.0, R Studio, and Chiplot (https://www.chiplot.online/). PCoA was performed for multiple sample type resistome comparison based on the Bray–Curtis dissimilarity matrix. Procrustes analysis was performed to investigate the relationship between microbial species and ARG compositions. The above analysis was conducted using the Vegan [93] and ggplot2 [94] packages in RStudio. Network analysis of ARGs and their bacterial hosts was conducted using Cytoscape v3.10.1. The number of reads supporting the links between ARGs and species was counted and used as the edge weights. Only links supported by ≥ 10 reads and species assigned with ≥ 5 ARGs were visualized. The phylogenetic trees and gene arrangements were visualized using tvBOT [95].

## Author Contributions

J.D., M.W., X.X., D.W., X.C., S.L., X.Z., Y.C., and Y.D. contributed to methodology. J.D., M.W., X.X., and D.W. performed the investigation. J.D. and M.W. contributed to formal analysis. J.D. contributed to visualization. L.L. and T.Z. contributed to supervision. J.D., L.L., and T.Z. contributed to writing the original draft. J.D., M.W., X.X., D.W., X.C., S.L., X.Z., Y.C., Y.D., T.T.Y.L., L.L., and T.Z. contributed to writing, reviewing, and editing.

## Conflicts of Interest

The authors declare no conflicts of interest.

## Supporting information




**Supporting File 1**: advs73551‐sup‐0001‐SuppMat.docx.

**Supporting File 2**: advs73551‐sup‐0002‐Supporting information II.xlsx.

## Data Availability

All sequencing data generated in this study have been deposited in the NCBI Sequence Read Archive (SRA) under BioProject accession code PRJNA1207805. The analysis scripts used in this study are available at https://github.com/dingjiahui123/Sewage_ARG.git.
