# Supplementary material for: Highly Resolved Community Sewage Metagenomics Unveiling Landscape and Transmission Patterns of Antibiotic Resistome in Hong Kong Populations
Source: Adv Sci (Weinh). 2026 Feb 27;13(20):e08389. doi: 10.1002/advs.202508389 (PMC13067863; doi:10.1002/advs.202508389)
Supplement: Supplementary file 1 — Supporting File 1: advs73551‐sup‐0001‐SuppMat.docx. [file ADVS-13-e08389-s001.docx]

Supporting Information I (SI1) for

**Highly resolved community sewage metagenomics unveiling landscape and transmission patterns of antibiotic resistome in Hong Kong populations**

Jiahui Ding^1^, Mengying Wang^1^, Xiaoqing Xu^1^, Dou Wang^1^, Xi Chen^1^, Shuxian Li^1^, Xiawan Zheng^1^, You Che^4^, Yu Deng^1,5^, Tommy T.Y. Lam^4,6,7,8^, Liguan Li^1,2*^, Tong Zhang^1,3,9,10,11*^

**Affiliations**

^1^ Environmental Microbiome Engineering and Biotechnology Lab, Center for Environmental Engineering Research, Department of Civil Engineering, The University of Hong Kong, Pokfulam Road, Hong Kong SAR, China.

^2^ Department of Science and Environmental Studies, The Education University of Hong Kong, 10 Lo Ping Road, Tai Po, New Territories, Hong Kong SAR, China.

^3^ School of Public Health, The University of Hong Kong, Pokfulam Road, Hong Kong SAR, China.

^4^ HKU-Pasteur Research Pole, The University of Hong Kong, Sassoon Road, Hong Kong SAR, China.

^5^ Faculty of Dentistry, The University of Hong Kong, Pokfulam Road, Hong Kong SAR, China.

^6^ State Key Laboratory of Emerging Infectious Diseases, School of Public Health, The University of Hong Kong, Sassoon Road, Hong Kong SAR, China.

^7^ Centre for Immunology & Infection, 17W Hong Kong Science & Technology Parks, Hong Kong SAR, China.

^8^ The Hong Kong Jockey Club Global Health Institute, Hong Kong SAR, China.

^9^ Department of Environmental Science and Engineering, Macau University of Science and Technology, Macao SAR, China.

^10^ State Key Laboratory of Marine Environmental Health, City University of Hong Kong, Hong Kong SAR, China.

^11^ Shenzhen Institute of Research and Innovation, The University of Hong Kong, Shenzhen, China.

*Corresponding author.

E-mail: [zhangt@hku.hk](mailto:zhangt@hku.hk); [lilig@eduhk.hk](mailto:lilig@eduhk.hk)

# Phone: +852-2857 8551; Fax: +852-2859 5337

Number of pages: 13

Number of Tables: 1

Number of Figures: 14

**Supplementary tables**

**Table S1. Statistics of Illumina and Nanopore sequencing data.** The information was provided in a separate Excel Table.

**Table S2.** **The assembly statistics of short- and long-read metagenomes.** The information was provided in a separate Excel Table.

**Table S3. Genetic location of major ARGs predicted from long reads generated by Nanopore sequencing.**

| ARG types | Total ARGs number | Plasmid carrying | Chromosome carrying |
| --- | --- | --- | --- |
| Aminoglycoside | 179,974 | 84,648 (47.03%) | 95,326 (52.97%) |
| Bacitracin | 114,084 | 20,922 (18.34%) | 93,162 (81.66%) |
| Beta-lactam | 181,696 | 73,455 (40.43%) | 108,241 (59.57%) |
| Chloramphenicol | 23,094 | 8,185 (35.44%) | 14,909 (64.56%) |
| MLS | 288,438 | 64,854 (22.48%) | 223,584 (77.52%) |
| Multidrug | 701,632 | 176,660 (25.18%) | 524,972 (74.82%) |
| Mupirocin | 25,455 | 3 (0.01%) | 25,452 (99.99%) |
| Polymyxin | 156,495 | 41,270 (26.37%) | 115,225 (73.63%) |
| Quinolone | 68,034 | 38,403 (56.45%) | 29,631 (43.55%) |
| Sulfonamide | 30,750 | 23,112 (75.16%) | 7,638 (24.84%) |
| Tetracycline | 268,535 | 75,896 (28.26%) | 192,639 (71.74%) |
| Trimethoprim | 41,211 | 17,670 (42.88%) | 23,541 (57.12%) |
| Vancomycin | 10,562 | 56 (0.53%) | 10,506 (99.47%) |

**Supplementary figures**


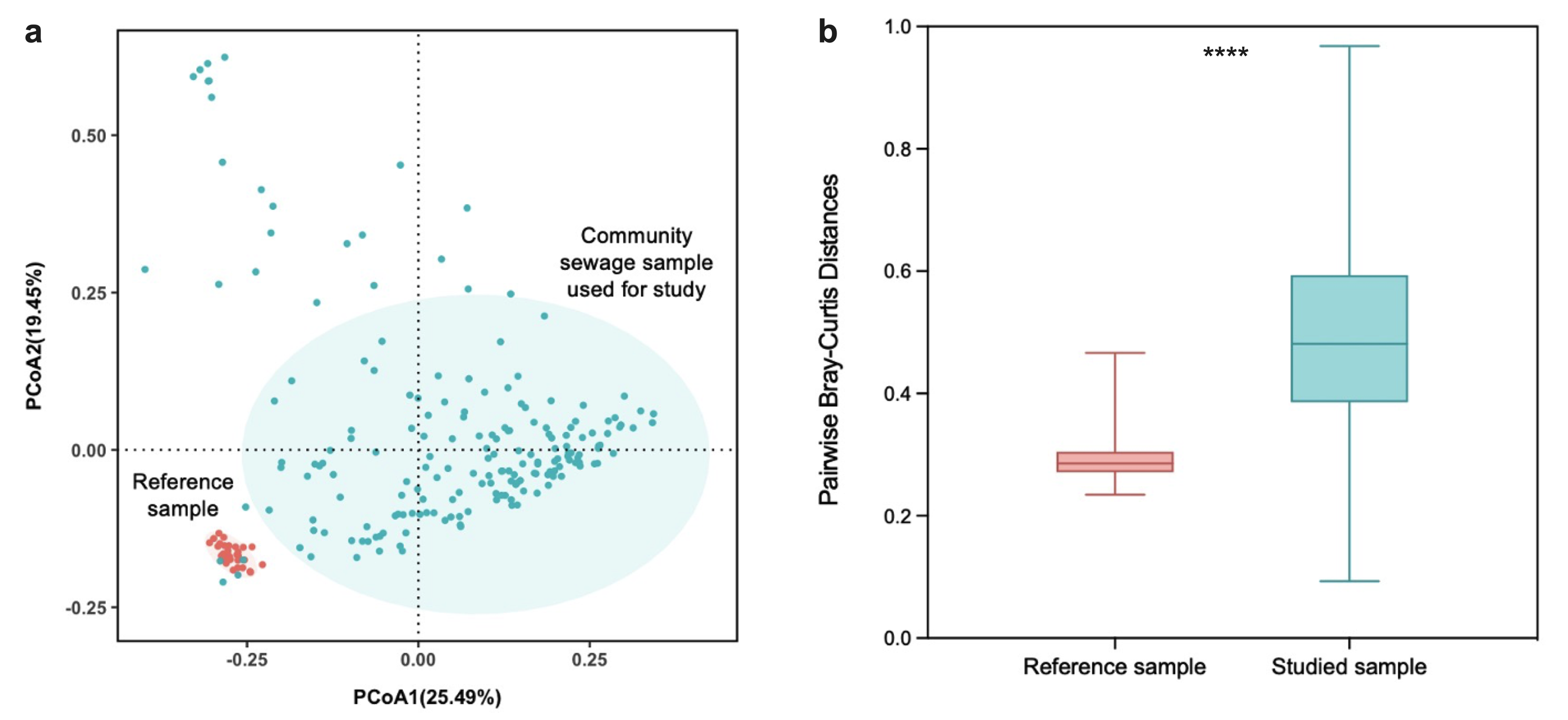


**Fig. S1. Reproducibility of the WWTP influent reference samples.** Principal coordinates analysis (PCoA) of the reference samples and community sewage samples based on Bray-Curtis distance of ARG composition at the ARG reference gene level (a). Colors denoted sample types. Boxplot showing variability determined by pairwise Bray-Curtis distance in ARG composition (b). Mann–Whitney U tests were conducted, and p-values less than 0.05 were considered statistically significant.


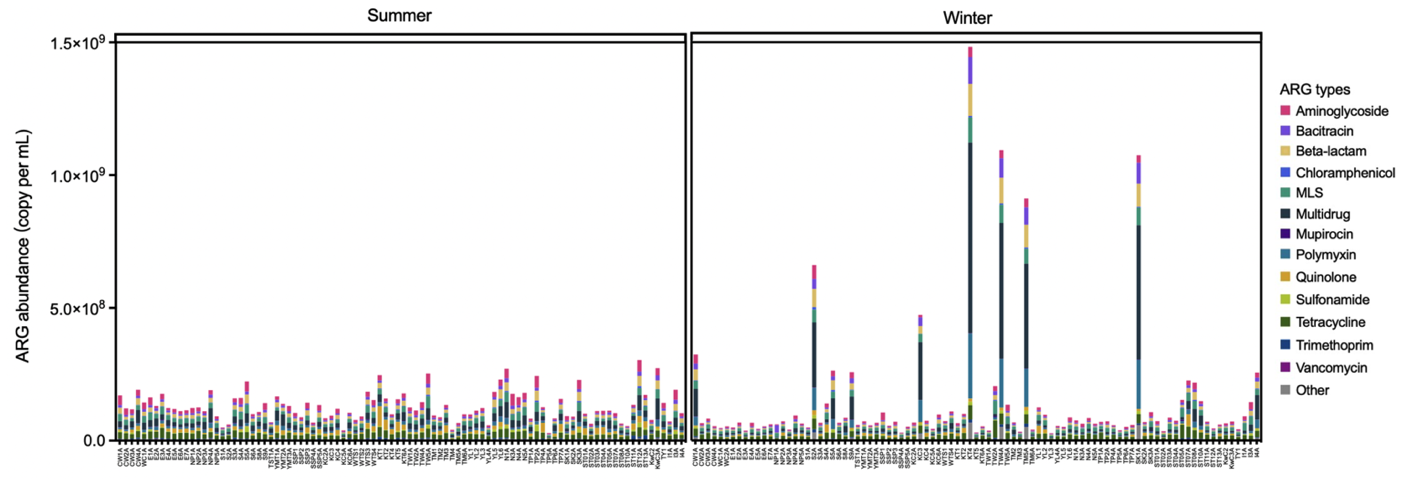


**Fig. S2. Concentrations of ARG types in the community sewage.** MLS was short for macrolide-lincosamide-streptogramin.


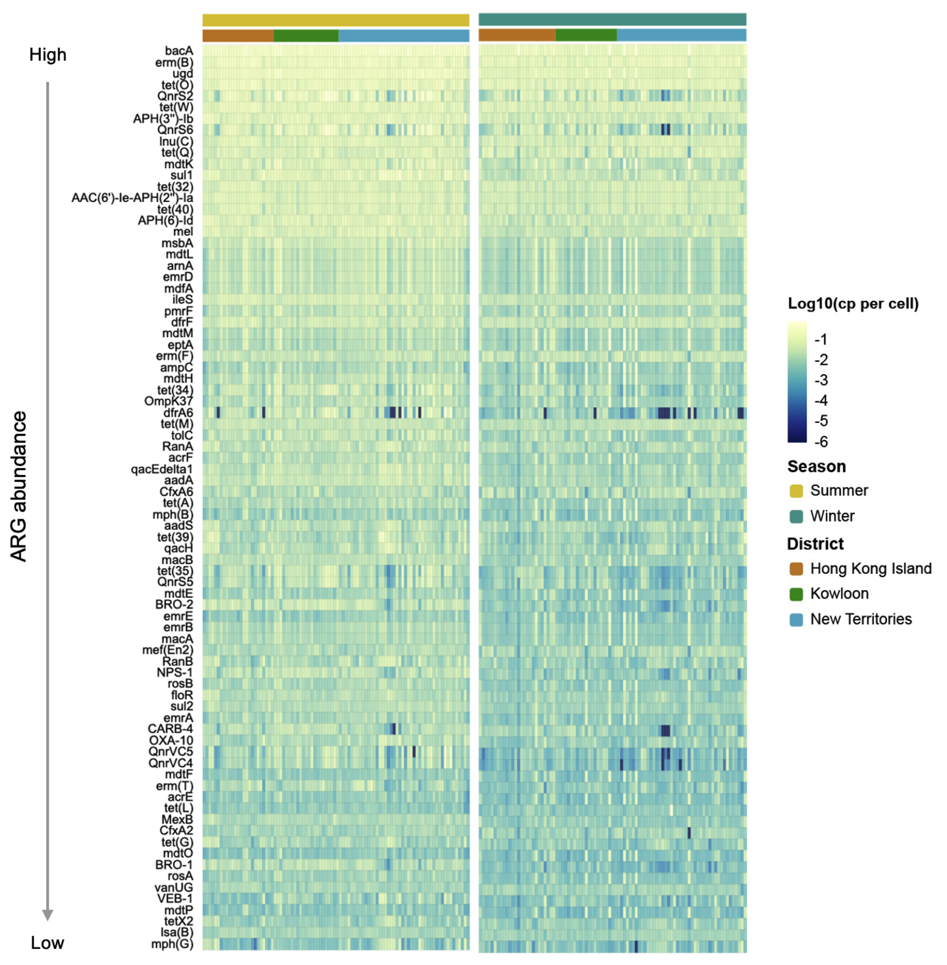


**Fig. S3. The mean abundance (log_10_) of 60 most abundant ARGs.**


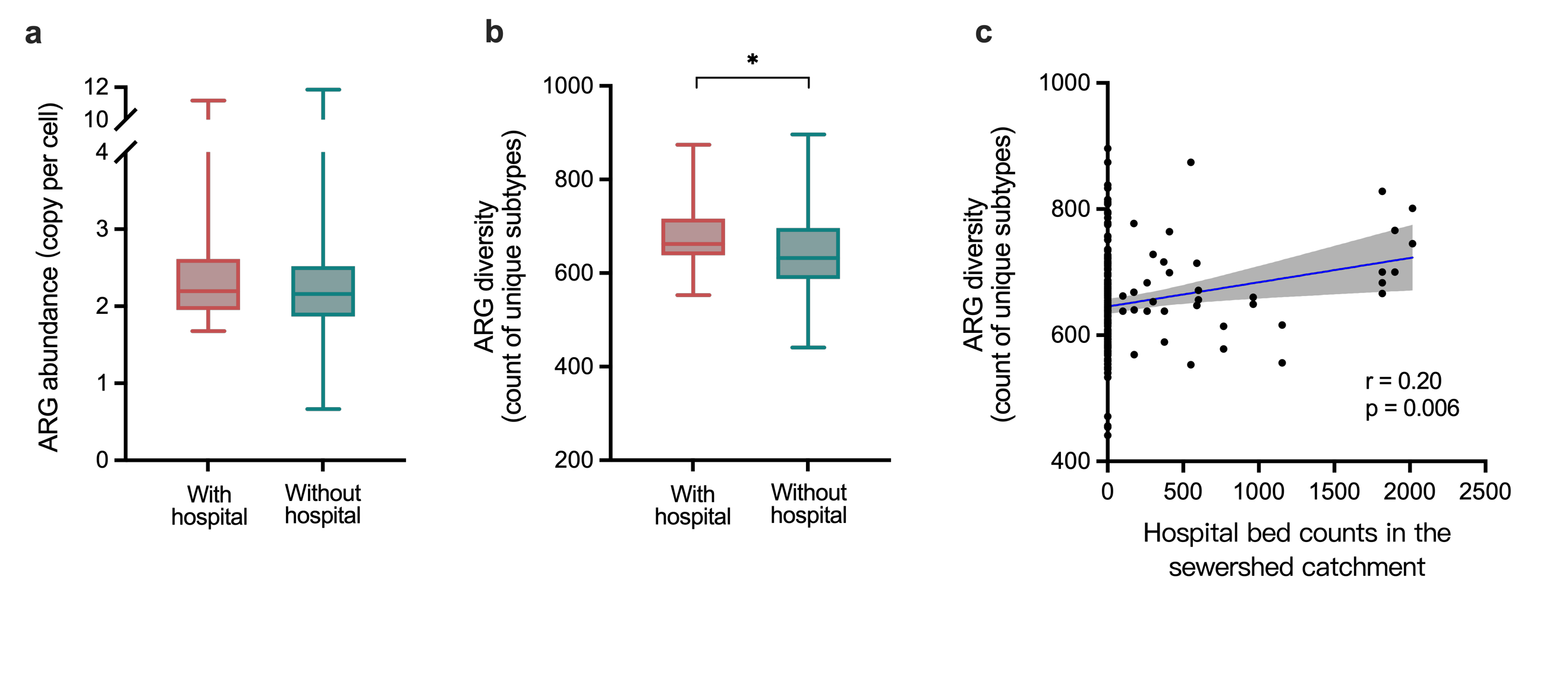


**Fig. S4. Effect of presence of hospitals in the sewershed catchment on the community sewage resistome.** **a.** ARG abundance and **b.** ARG diversity in the community sewage with or without hospitals covered in the sewershed catchment. Mann–Whitney U tests were conducted, and p-values less than 0.05 were considered statistically significant. **c.** Correlation analysis between hospital beds in the sewershed catchment and ARG diversity. The hospital bed counts were obtained from Hong Kong’s hospital authority (https://www.ha.org.hk/).


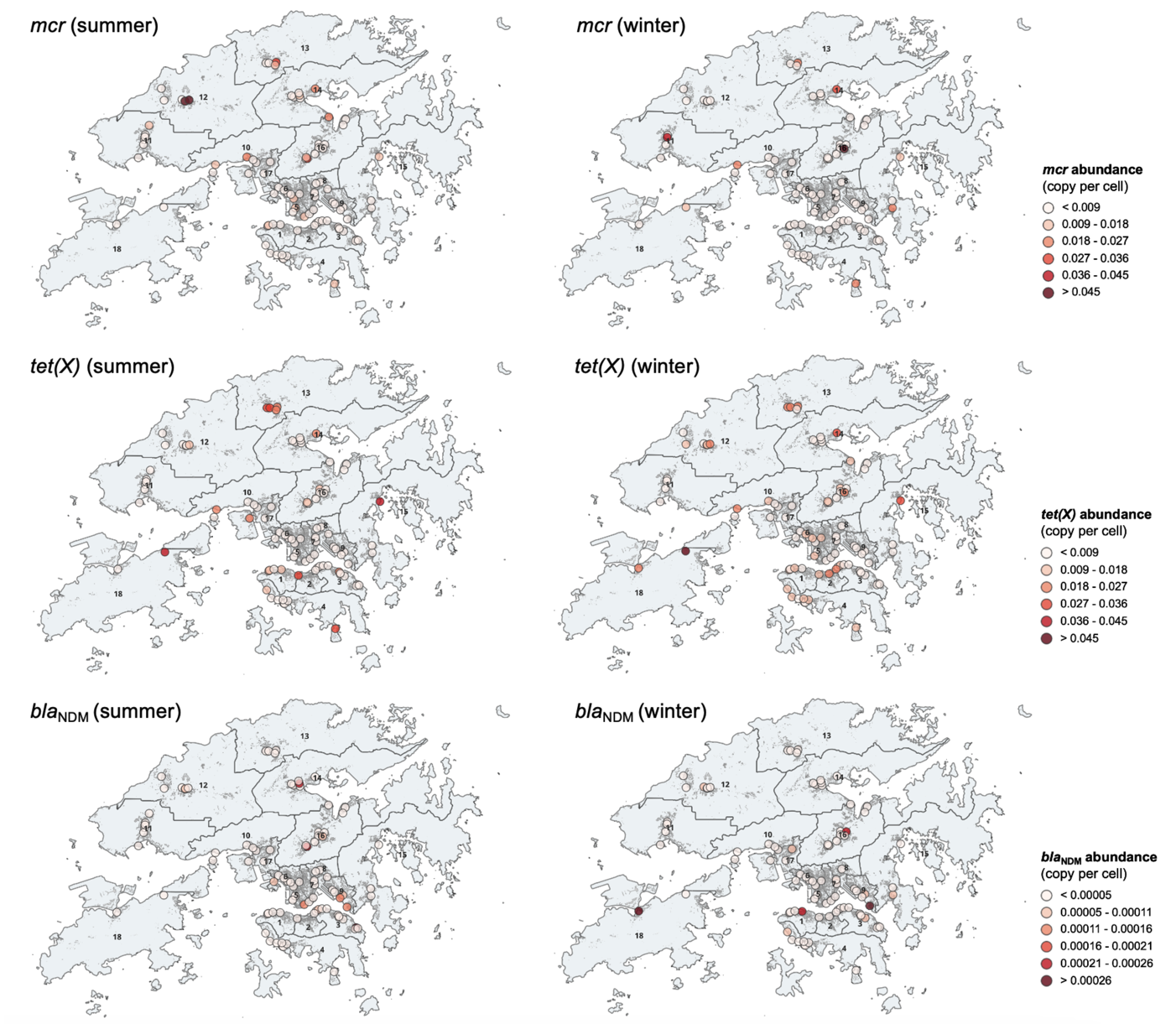


**Fig. S5. Spatial distribution and seasonal variation of ARGs in Hong Kong community sewage.** Only the abundance of *mcr*, *tet*(X), and *bla*_NDM_ were visualized. The unit is copies of ARGs per cell. Residential areas across Hong Kong were indicated in grey shade.


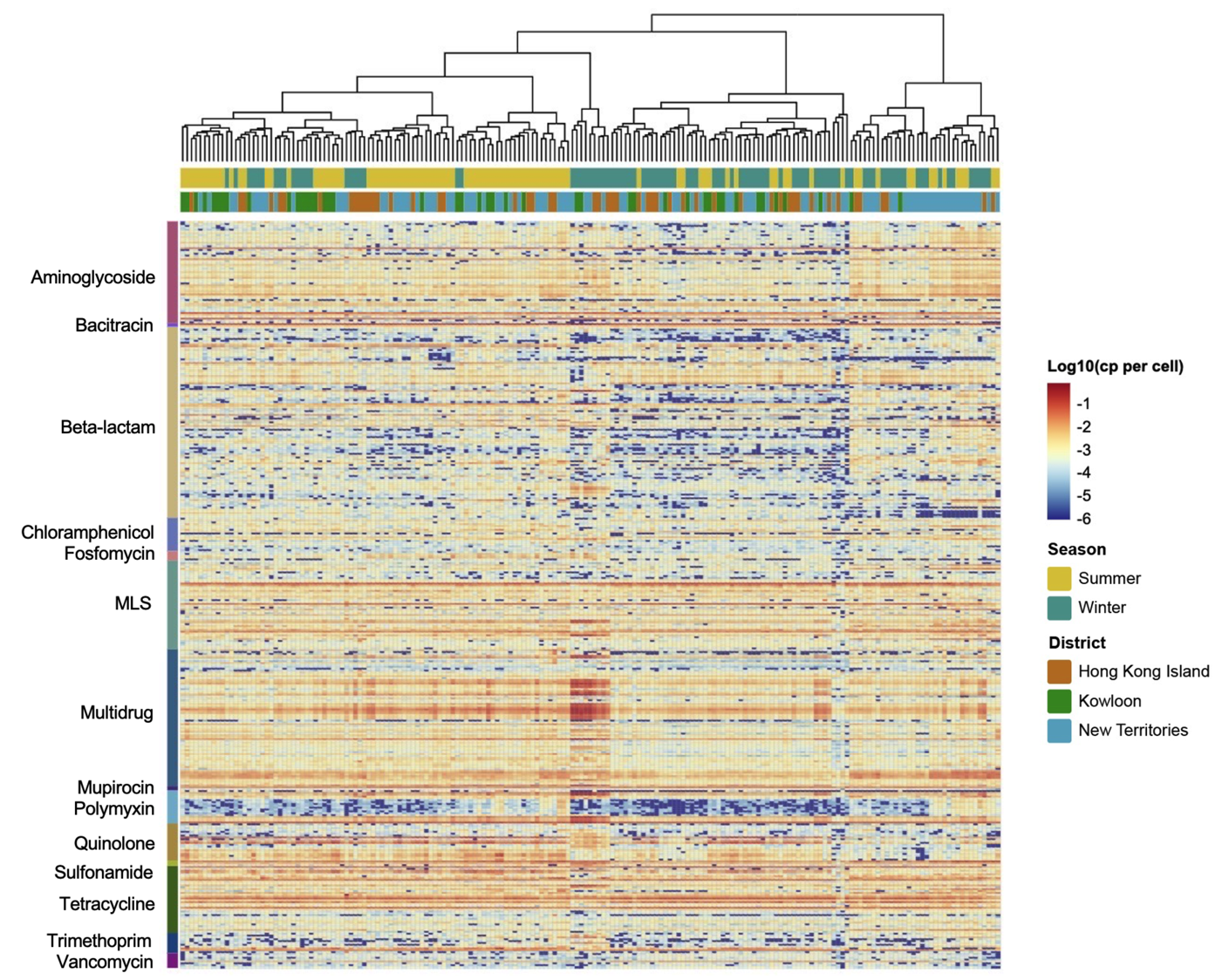


# Fig. S6. Heatmap and hierarchical clustering showing the resistome profiles at ARG subtype level. ARG abundances were transformed to log_10_ scale. The dendrogram was created based on Euclidean distance with ward.D2 clustering method.

#
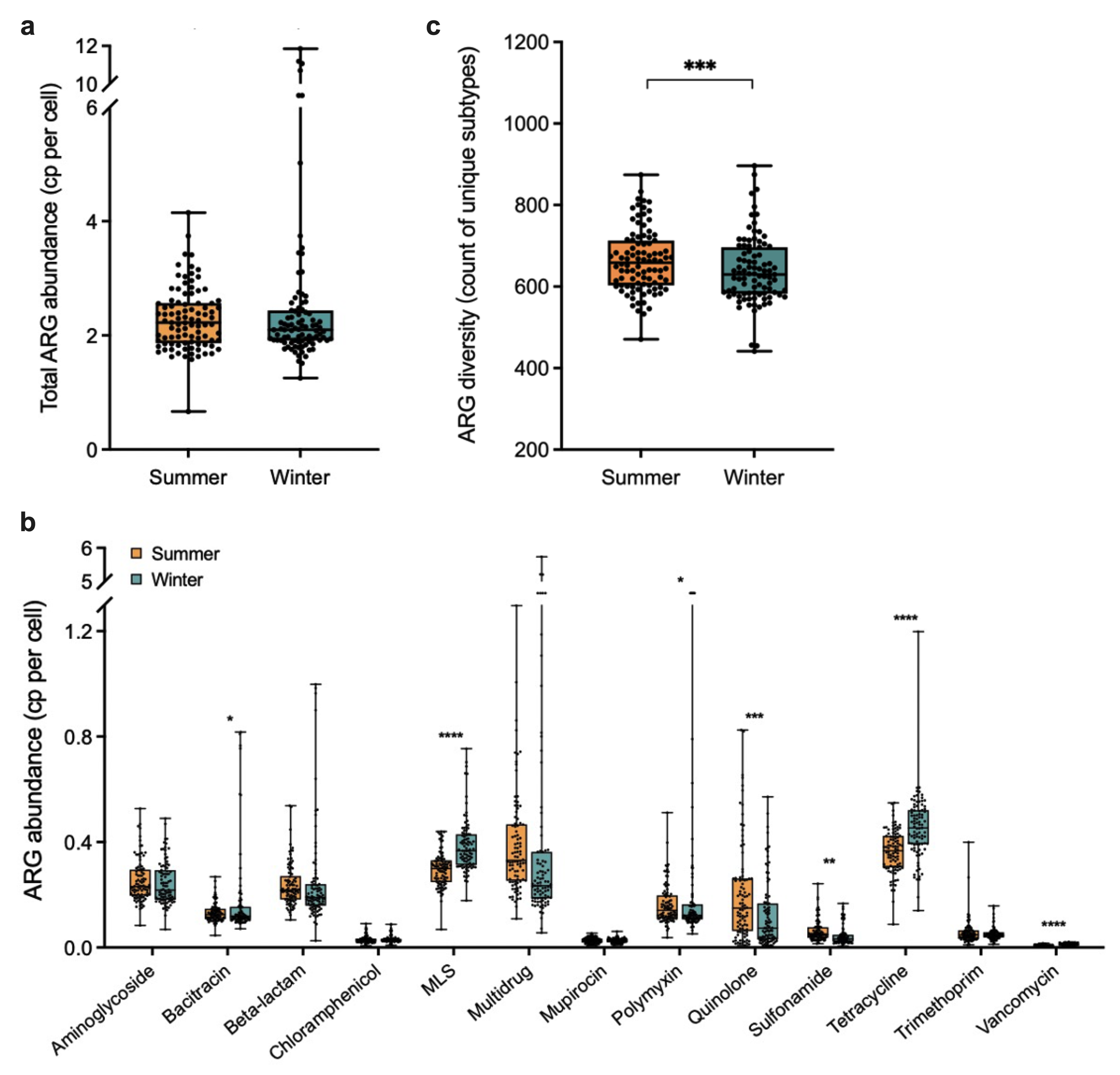


**Fig. S7. Seasonal variation of ARGs between summer and winter. a.** Total ARG abundance. **b.** ARG subtype abundance. **c.** ARG diversity. Wilcoxon signed rank tests were conducted, and p-values less than 0.05 were considered statistically significant.


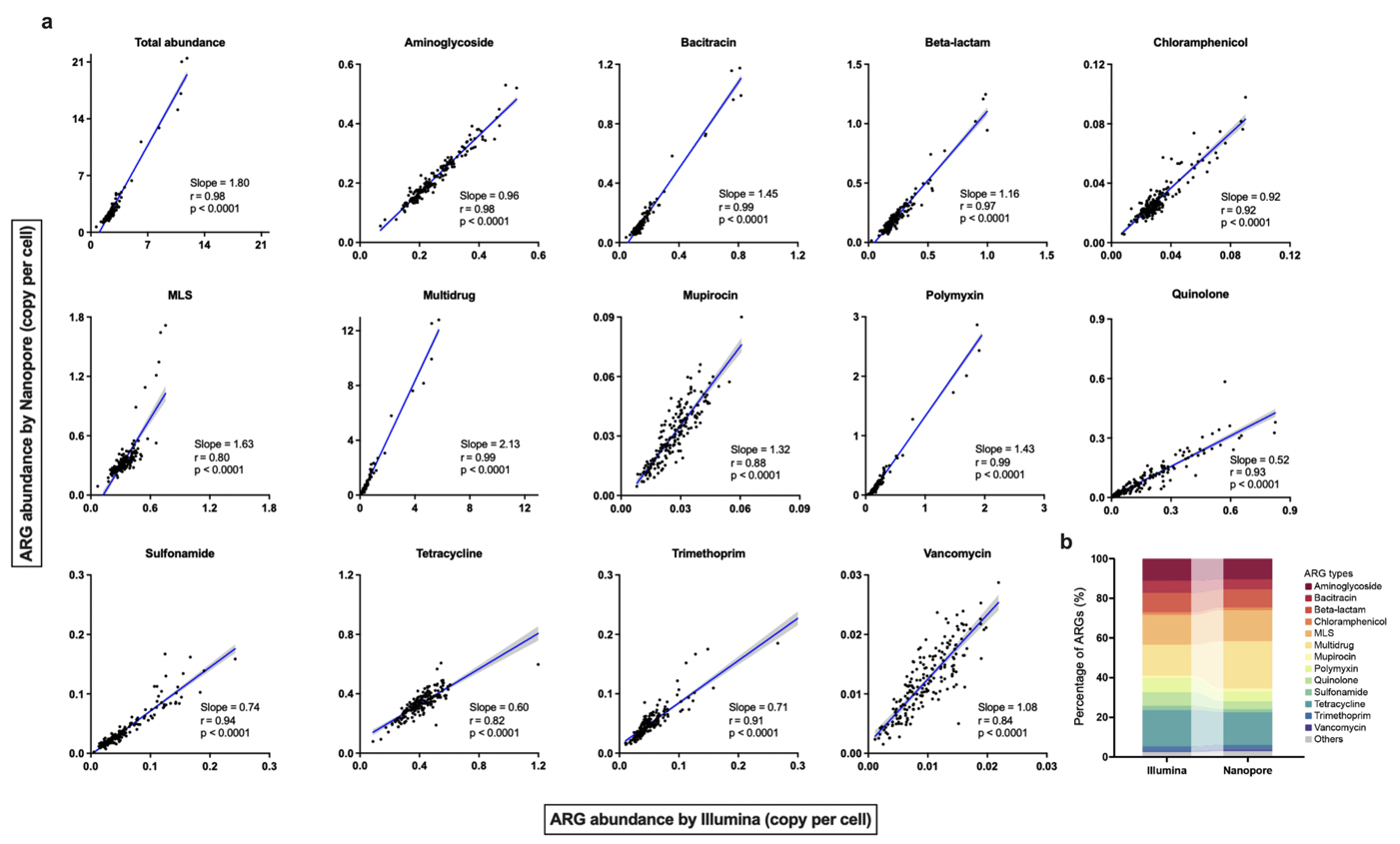


**Fig. S8. Comparison of resistome profiles obtained by Illumina and Nanopore metagenomic sequencing.** **a.** Correlation analysis of major ARG abundances in copies of ARGs per cell obtained from the two platforms across all samples. **b.** Stacked bar plots showing the mean percentages of ARG types calculated by the two datasets.


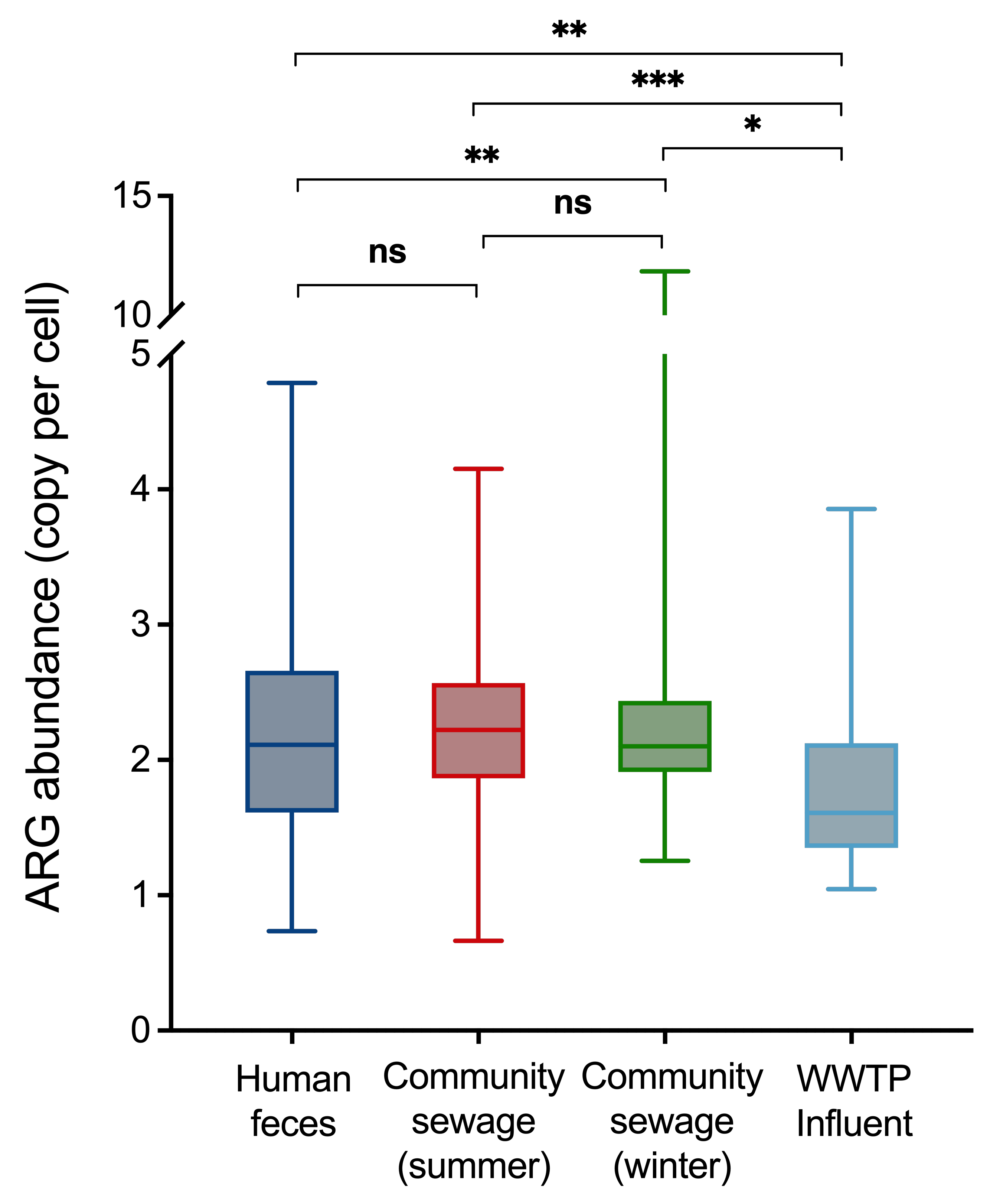


**Fig. S9. Comparison of ARG abundances in human feces and sewage metagenomes of Hong Kong.** Mann–Whitney U tests were conducted, and p-values less than 0.05 were considered statistically significant.


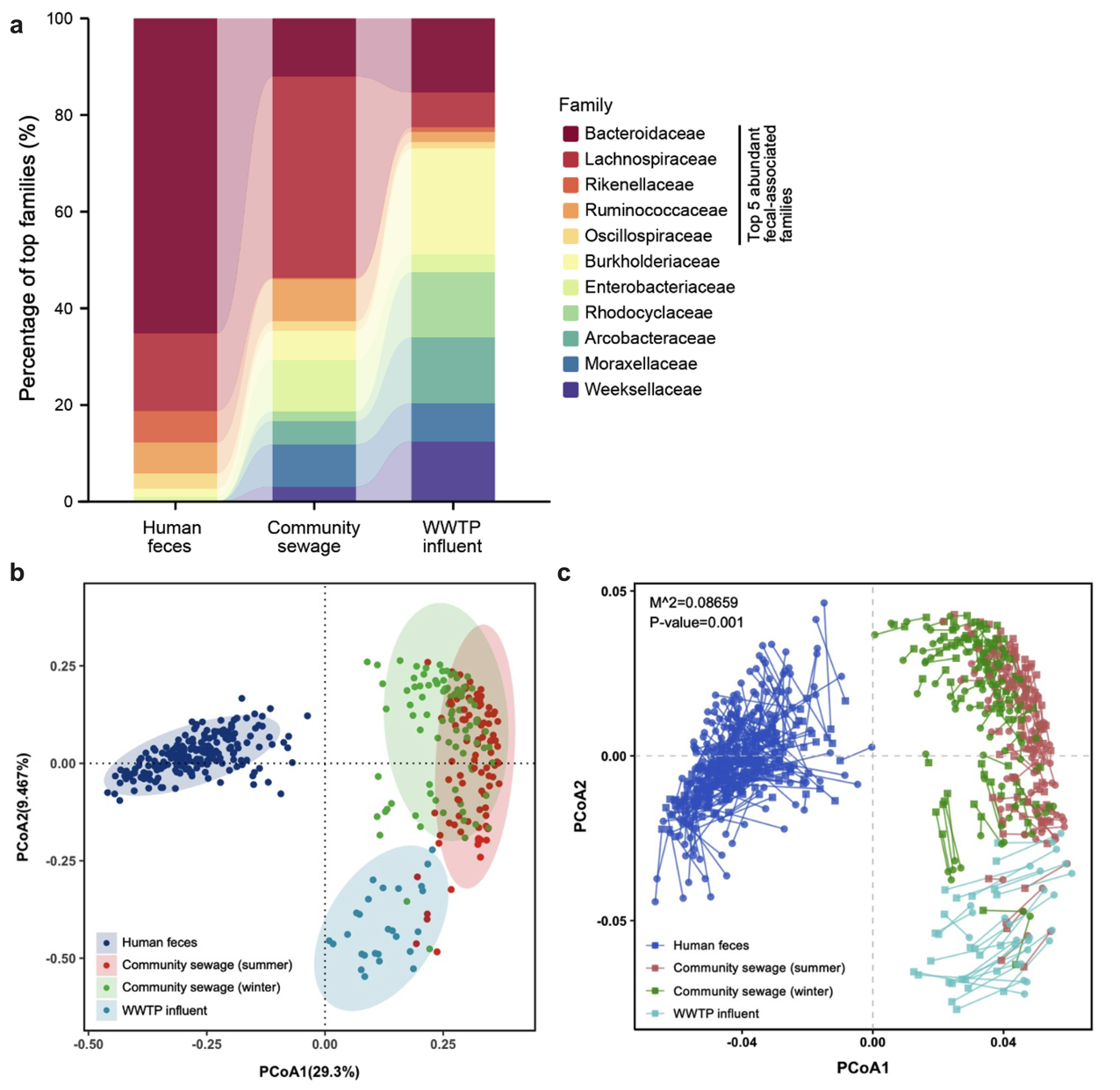


**Fig. S10.** **Comparison of microbial compositions between human feces and sewage. a.** Stacked bar plots showing the most abundant families (top 5 from each dataset) in human feces, community sewage, and WWTP influent. Percentage was the proportion of specific families among the abundant families visualized. **b.** PCoA of overall bacterial communities based on the Bray-Curtis dissimilarity matrix at the species level. Colors denoted sample types. **c.** Correlation of microbial species and ARG compositions across different sample types performed by Procrustes analysis. The circle end of the lines represented the resistome position, and the square end of the lines represented the taxonomy position. M^2^ and p*-*value were shown to indicate the robustness of the correlation.


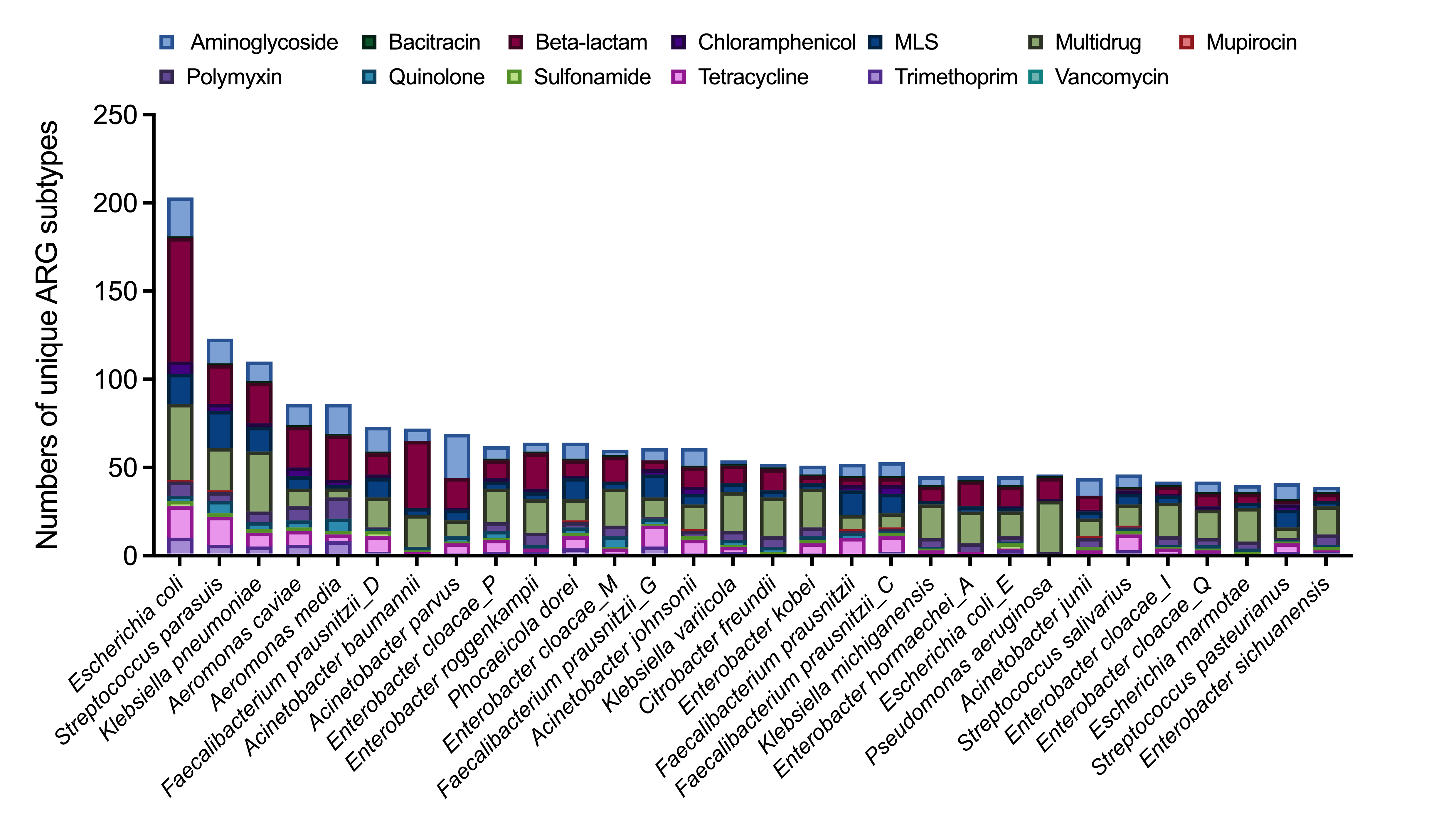


**Fig. S11. The ARG diversity among potentially pathogenetic species.** To facilitate viewing, only the top 30 pathogens that harbored the most diverse ARGs were shown. Different ARG types were indicated in different colors.


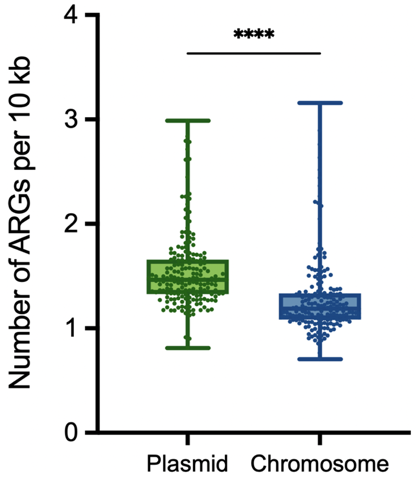


**Fig. S12. Numbers of ARGs per 10 kb plasmid and chromosome sequences in the community sewage.** Wilcoxon signed rank tests were conducted, and p-values less than 0.05 were considered statistically significant.


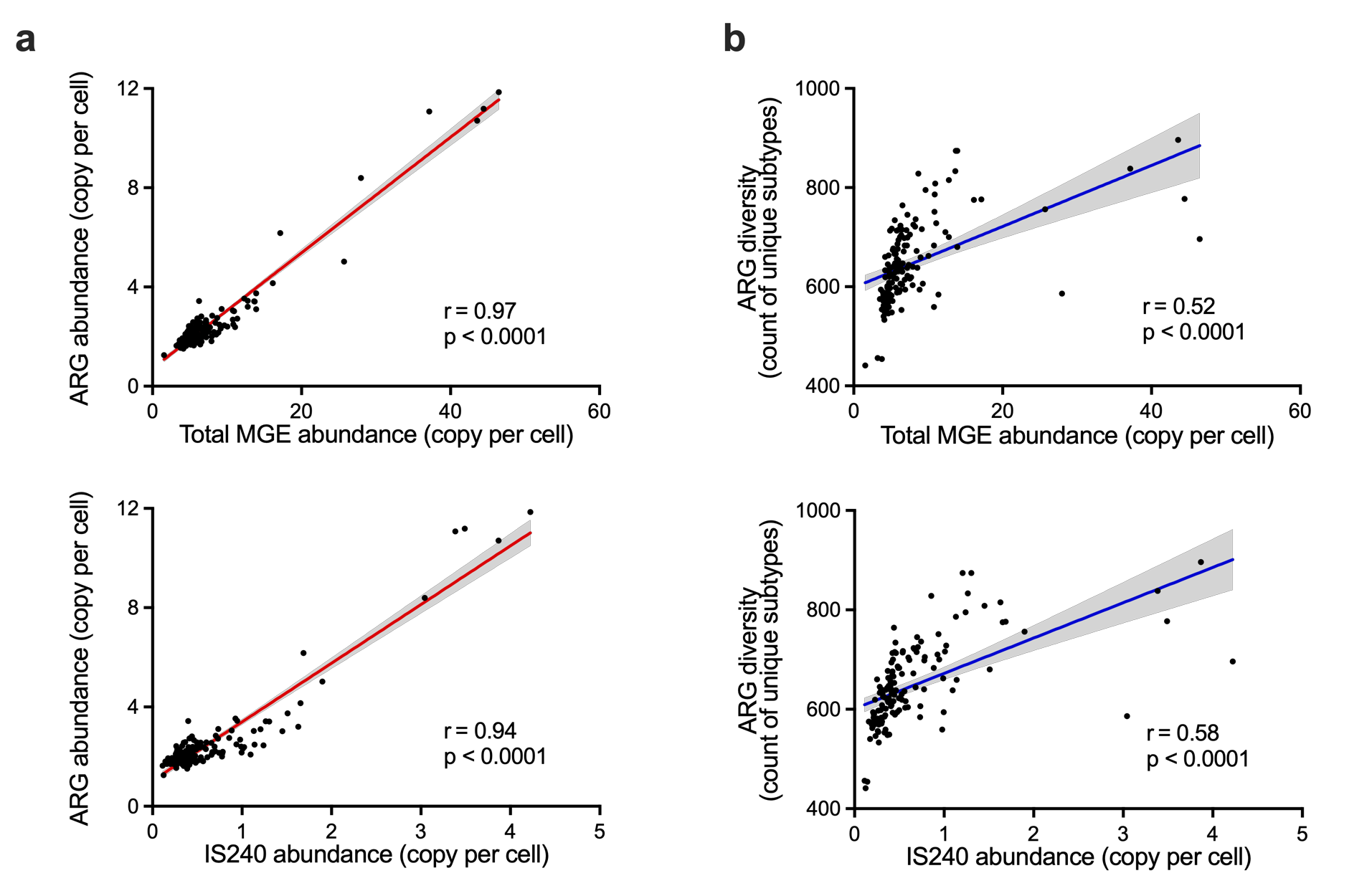


**Fig. S13. Correlations of ARG abundances (a) and ARG diversity (b) with MGEs across all samples.** Each point represented one sample. Total MGE abundance and the abundance of IS240 were presented.


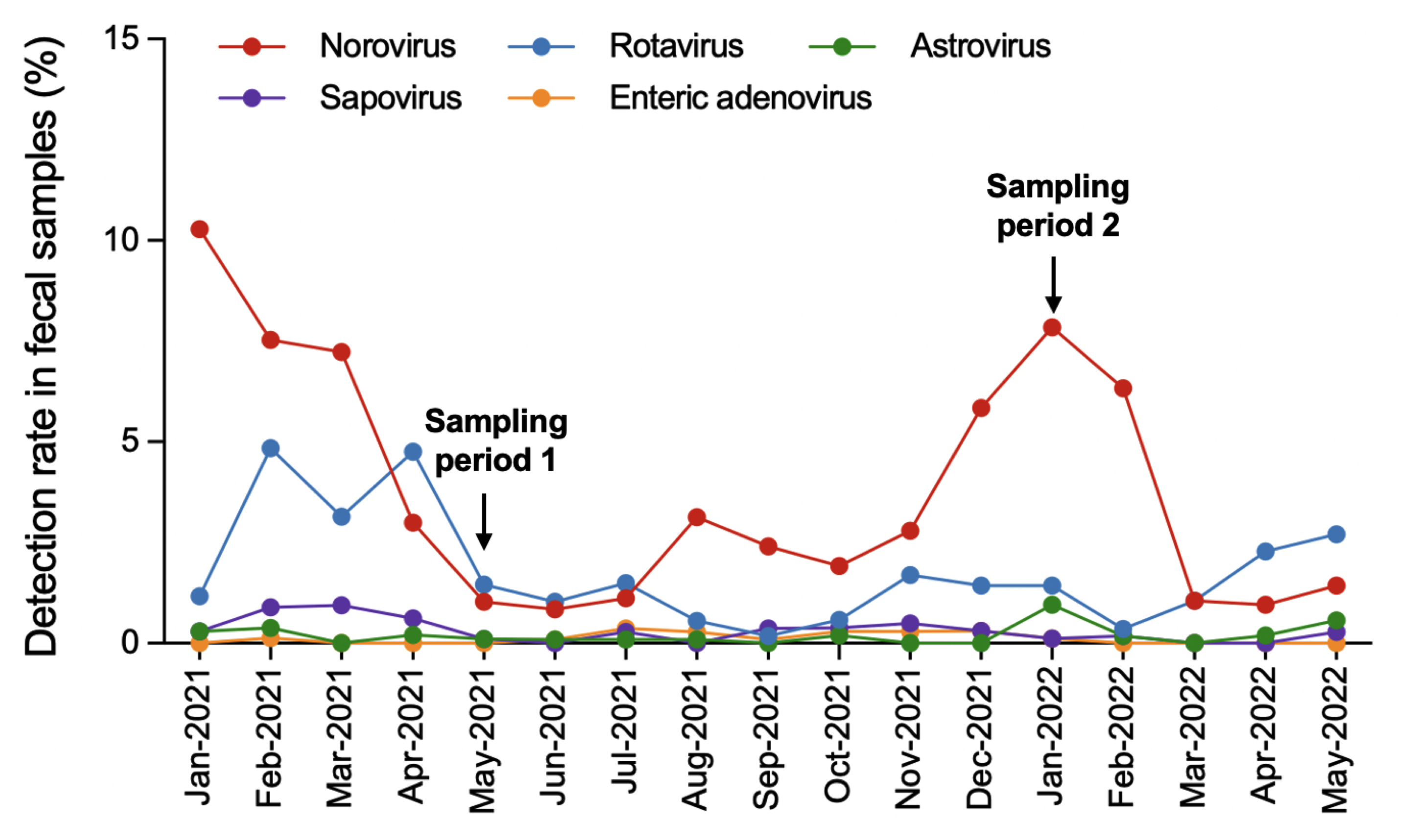


**F****ig. S14.** **Clinical surveillance for gastroenteritis viruses in Hong Kong.** The monthly detection rates were obtained from Hong Kong’s Centre for Health Protection (CHP) (https://www.chp.gov.hk/), which was derived from clinical testing of 472–1,088 fecal samples per month.
